# ETNet: an interpretable transformer framework for enhancer–enhancer interaction prediction with cross-context transferability

**DOI:** 10.1093/bib/bbaf634

**Published:** 2025-11-30

**Authors:** Shuaibin Wang, Tong Chen, Zhongxin Yang, Zhen Liang, Yin Shen

**Affiliations:** School of Biomedical Engineering, Anhui Medical University, No. 81 Meishan Road, Shushan District, Hefei 230032, Anhui, China; School of Biomedical Engineering, Anhui Medical University, No. 81 Meishan Road, Shushan District, Hefei 230032, Anhui, China; School of Biomedical Engineering, Anhui Medical University, No. 81 Meishan Road, Shushan District, Hefei 230032, Anhui, China; School of Biomedical Engineering, Anhui Medical University, No. 81 Meishan Road, Shushan District, Hefei 230032, Anhui, China; The Affiliated Chuzhou Hospital of Anhui Medical University (The First People's Hospital of Chuzhou), No. 369 Zuiweng West Road, Nanqiao District, Chuzhou 239001, Anhui, China; School of Biomedical Engineering, Anhui Medical University, No. 81 Meishan Road, Shushan District, Hefei 230032, Anhui, China; Second Affiliated Hospital of Anhui Medical University, No. 678 Furong Road, Economic and Technological Development Zone, Hefei 230601, Anhui, China

**Keywords:** enhancer–enhancer interactions, transformer architecture, transfer learning, regulatory genomics, model interpretability

## Abstract

Enhancer–enhancer interactions (EEIs) are critical regulatory components in transcriptional networks but remain computationally challenging to predict. While enhancer–promoter interactions have been extensively studied, EEIs remain comparatively underexplored. We developed ETNet (Enhancer–enhancer Interaction Explainable Transformer Network), a deep learning architecture integrating convolutional neural networks with Transformer modules to predict EEIs from DNA sequences. Evaluation across three cell lines (GM12878, K562, MCF-7) demonstrated superior performance compared to existing methods including EnContact, with statistical significance confirmed through DeLong tests across six cell lines. Rigorous validation through cross-validation and enhancer-level data partitioning confirmed robust generalization. ETNet exhibited effective cross-cell type transfer learning and showed transferability to enhancer–promoter interaction tasks, providing exploratory evidence for shared chromatin interaction principles. Feature attribution analysis recovered cell-type-specific regulatory motifs consistent with known transcription factors and revealed computational evidence for super-additive cooperative mechanisms, with cooperativity negatively correlating with sequence similarity—patterns representing hypothesis-generating observations requiring experimental validation. Proof-of-concept analysis demonstrated how single-nucleotide polymorphisms in JAK–STAT pathway genes may influence predicted interactions through motif alterations. ETNet advances computational approaches for studying enhancer interactions and provides a framework combining predictive capability with exploratory interpretability.

## Introduction

An extensive body of research has established the pivotal regulatory functions of *cis*-regulatory elements within the noncoding genome, particularly enhancers and promoters, in orchestrating transcriptional processes [1]. Enhancers establish long-range regulatory relationships with promoters through chromatin looping mechanisms while simultaneously forming complex regulatory networks with other enhancers to coordinate gene expression. Despite substantial genomic distances separating enhancers and promoters in the linear genome, these elements establish functional connections through physical interactions within the 3D chromatin architecture. Moreover, distinct enhancers can coalesce to form synergistic regulatory modules through chromatin contacts. These enhancer–enhancer interactions (EEIs) represent fundamental components of gene regulatory networks with profound implications for transcriptional dynamics [2, 3]. During cardiac lineage specification, enhancers exhibit stage-specific activity patterns and form dynamic interaction networks coordinating gene expression across developmental transitions. Through cooperative interactions on shared target genes, multiple enhancers can achieve synergistic transcriptional activation, contributing to robust expression of lineage-determining genes. For instance, coordinated activity of transcription factors such as MEF2C, Hand2, and Akt1 at enhancer clusters during cardiac reprogramming indicates the formation of integrated regulatory networks. Disruption of such coordinated enhancer function may contribute to developmental defects or disease states, highlighting the importance of understanding EEI mechanisms [4].

To investigate the physical interactions between distant regulatory elements within chromatin architecture, researchers have developed various experimental methodologies, including chromosome conformation capture (3C) and its derivatives (4C, 5C), as well as chromatin interaction analysis by paired-end tag sequencing (ChIA-PET) [5, 6]. Recent technological advances in genome-wide approaches, such as Hi-C, Capture Hi-C, and HiChIP, have enabled the generation of comprehensive 3D chromatin interaction maps with unprecedented resolution [7]. However, these experimental techniques typically require exceptionally high sequencing depth and encounter significant limitations when applied across diverse cellular contexts. Consequently, the development of computationally efficient, cost-effective, and reliable predictive methods has become increasingly imperative for elucidating the intricate complexity of long-range enhancer interaction networks [8]. In recent years, significant advances have been made in applying machine learning and deep learning methodologies to predict chromatin interactions, encompassing enhancer–promoter interactions (EPIs), promoter–promoter interactions (PPIs), and protein-mediated chromatin loops. Pioneering computational models including TargetFinder, Sequence-based Promoter-Enhancer Interaction with Deep learning (SPEID), deepTACT, and deepPHiC have effectively employed convolutional neural networks (CNNs), recurrent neural networks (RNNs), and attention mechanisms to extract discriminative features from DNA sequences and epigenetic data, thereby substantially improving prediction accuracy for regulatory interactions [9–12]. Building upon these foundational approaches, more sophisticated methodologies have subsequently emerged, which can be broadly categorized into sequence-based and graph-based paradigms. Sequence-based methods such as EPInformer integrate CNNs with Transformer architectures to directly process DNA sequences, while graph-based methods such as GATv2EPI employ graph neural networks to model regulatory interactions as network structures. Although both paradigms offer valuable insights, they operate on fundamentally different input representations, with sequence-based approaches analyzing nucleotide patterns and graph-based approaches requiring preconstructed network topologies [13, 14]. These progressive refinements in computational approaches have equipped researchers with increasingly powerful analytical frameworks for deciphering the intricate complexity of gene regulatory networks and their 3D organization within the nucleus.

Despite substantial research progress on EPIs, computational approaches for EEI prediction remain comparatively limited. To date, EnContact represents the primary dedicated deep learning method for EEI prediction [15], employing attention mechanisms to identify interacting enhancer pairs. While the extensive methodological advances in EPI prediction provide valuable conceptual frameworks—including CNNs for local sequence feature extraction, Transformers for long-range dependency modeling, and attention mechanisms for learning nonlinear combinatorial effects—the specific regulatory logic underlying EEIs requires dedicated investigation. Experimental evidence has demonstrated that enhancers frequently establish simultaneous contacts with multiple other enhancers and promoters, thereby orchestrating coordinated regulation of gene expression networks [16]. Notably, the combined regulatory effects of enhancer pairs typically exhibit near-additive or super-additive properties, wherein their aggregate impact on promoter activity can exceed the sum of their individual contributions. This super-additive effect manifests more prominently when enhancers associate with weaker promoters and appear attenuated when linked to stronger promoters. These differential interaction patterns suggest that enhancer–enhancer regulatory mechanisms may operate through distinct molecular pathways contingent upon promoter strength, thus serving critical functions in gene coregulation, cellular differentiation processes, and the development of pathological conditions [17, 18].

Building upon existing research, we present ETNet (Enhancer–enhancer Interaction Explainable Transformer Network), a deep learning framework integrating CNNs with Transformer-based modules. ETNet makes several key contributions: (i) It achieves robust predictive performance across three cell lines (GM12878, K562, MCF-7) with statistical validation across six cell lines, confirming significant improvements over EnContact. (ii) It demonstrates effective cross-cell type transfer learning through selective fine-tuning and shows cross-task transferability to EPI prediction as an exploratory investigation of shared regulatory principles. (iii) Through feature attribution analysis using DeepLIFT (Deep Learning Important FeaTures), it recovers cell-type-specific regulatory motifs and provides computational evidence for super-additive cooperative mechanisms—findings requiring experimental validation. (iv) It demonstrates proof-of-concept analysis of how Single-nucleotide polymorphisms (SNPs) in JAK–STAT pathway genes may alter predicted interactions, providing a framework for variant prioritization. These contributions advance computational approaches for studying enhancer interactions and establish a framework combining predictive capability with exploratory interpretability.

## Material and Methods

### Construction and processing of enhancer–enhancer interaction datasets

#### Cell line selection and data sources

In this study, we analyzed chromatin interaction loops using ChIA-PET data processed through the ChINN methodology [19]. We selected GM12878, K562, and MCF-7 cell lines to balance biological diversity with data availability and quality. These cell lines represent distinct cellular contexts: GM12878 (B lymphoblastoid cells, near-normal physiological state), K562 (myeloid leukemia), and MCF-7 (breast epithelial cancer) (Supplementary Table 1). This selection encompasses both hematopoietic and epithelial lineages, as well as normal and malignant conditions, providing diverse chromatin architectures and enhancer regulatory landscapes for comprehensive evaluation of model generalizability. All three cell lines possess high-quality multi-omics datasets in public repositories such as ENCODE, including Hi-C, ChIA-PET, ATAC-seq, ChIP-seq, and gene expression data. This data richness enables rigorous model training and validation while minimizing biases from incomplete or inconsistent annotations, allowing thorough assessment of ETNet’s transferability across cell-type-specific regulatory features and pathological conditions.

To further evaluate model generalizability across broader cellular diversity, we expanded our analysis to include three additional cell lines: IMR90 (fetal lung fibroblasts), HCT116 (colorectal carcinoma), and HCASMC (human coronary artery smooth muscle cells) (Supplementary Table 8). These additional cell lines extend tissue representation to mesenchymal and vascular lineages, providing complementary evaluation of model robustness across diverse chromatin contexts. The chromatin interaction data for IMR90, HCT116, and HCASMC were obtained from these three projects [20–22] and processed using identical protocols as the initial three cell lines. Statistical comparison between ETNet and EnContact across all six cell lines was performed using the DeLong test to assess significance of Area Under the Receiver Operating Characteristic Curve (AUC) differences.

#### Enhancer–enhancer interaction identification and dataset construction

**Figure 1:**
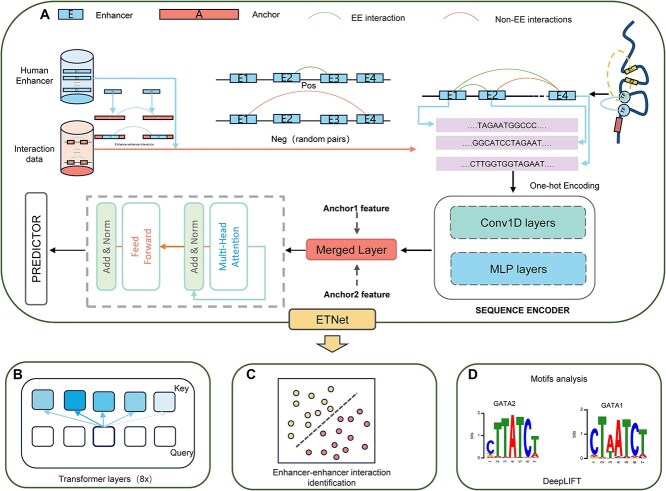
ETNet architecture and workflow. (A) Data processing pipeline showing sequential convolutional and transformer layers for hierarchical feature extraction from enhancer–enhancer interaction pairs; (B) multi-head attention mechanism with 8 parallel attention heads capturing long-range sequence dependencies; (C) binary classification module integrating extracted features for interaction prediction; (D) functional region identification through iterative perturbation analysis using greedy search with dinucleotide shuffling.

To systematically identify EEIs, we integrated these interaction loops with human permissive enhancers obtained from the FANTOM5 database [23]. Permissive enhancers are regulatory sequences that maintain potential activity across multiple tissues or cell types, characterized by positional flexibility and open chromatin states in diverse cellular contexts. Unlike robust enhancers, which exhibit high cell-type-specific activity, permissive enhancers represent latent regulatory capacity that can be activated under specific conditions. This selection strategy enables comprehensive evaluation of EEIs across diverse cellular environments and is particularly valuable for cross-cell-type transfer learning applications. For each chromatin interaction loop containing two anchors, we systematically identified overlapping enhancers from the FANTOM5 dataset. When an enhancer fully overlapped with either anchor, we retained that interaction while eliminating duplicates, thereby establishing our positive sample set of EEIs. Subsequently, we constructed a balanced negative sample set by randomly pairing enhancers from left and right anchors, explicitly excluding any combinations present in the positive set to avoid data contamination (Fig. 1). Statistical analysis of the FANTOM5 enhancer dataset revealed that the 95th percentile of enhancer length is 541 bp, indicating that most enhancers fall within a compact size range. To ensure comprehensive and consistent sequence coverage while capturing flanking regulatory context, all enhancer regions were standardized to 2000 bp centered on their midpoints. This window size has been widely adopted in enhancer analysis and interaction prediction studies, providing both methodological consistency and sufficient contextual information for feature extraction. The resulting dataset, comprising 34 482 EEI pairs, was stratified into training, validation, and testing sets at a ratio of 8:1:1.

### Validation strategies for information leakage control

To address potential information leakage from enhancer overlap between training and test sets, we implemented two complementary validation strategies. First, we performed 10-fold random cross-validation where the dataset was randomly partitioned into 10 folds, with each fold serving as the test set once, while the remaining 9 folds served as training data. Performance metrics were averaged across all 10 folds to assess model stability. Second, we implemented a dual-constraint enhancer-level splitting strategy to ensure complete separation of enhancers between training and test sets. This strategy enforces two constraints: (i) if an enhancer appears in any test set interaction pair, all interaction pairs containing that enhancer are excluded from the training set, and (ii) within the test set, each enhancer appears in at most one interaction pair through systematic deduplication. This approach eliminates any possibility of the same enhancer appearing in both training and test sets, providing a stringent evaluation of model generalization capability.

### Super-enhancer evaluation

To evaluate model performance on super-enhancer regulatory architecture, we obtained super-enhancer annotations for GM12878 (*n* = 257), K562 (*n* = 742), and MCF-7 (*n* = 117) from the dbSUPER database [24]. Each EEI pair was categorized based on its relationship to super-enhancers into three types: SE_intra (both enhancers located within the same super-enhancer), SE_inter (enhancers located in different super-enhancers), and SE_out (interactions not associated with any super-enhancer).

### Negative sampling strategy comparison

To evaluate potential bias from the random pairing approach for negative sample generation, we implemented two additional strategies for systematic comparison. The first strategy employs distance-based sampling where negative samples are generated by partitioning the genomic distance distribution of positive enhancer pairs into intervals and randomly sampling same-chromosome enhancer pairs from each interval to match the positive distribution [9]. The second strategy uses a side approach where one enhancer from a positive pair is retained while the partner is randomly shuffled, generating negative samples that preserve partial biological context while introducing controlled variation.

### DNA sequence encoding

DNA sequences were encoded using the one-hot encoding method [25, 26], a well-established approach in deep learning applications for genomic sequence analysis. This encoding schema systematically converts each nucleotide in a DNA sequence into a binary vector of length four, corresponding to the four possible nucleotide bases (A, C, G, T). Specifically, each nucleotide base is represented as follows: A = [1,0,0,0], C = [0,1,0,0], G = [0,0,1,0], and T = [0,0,0,1], while unknown or ambiguous bases (N) are represented as [0,0,0,0].


$$ R\in 0,{1}^{L\times 4} $$

This encoding methodology transforms the DNA sequence into a structured binary matrix *R*, where *L* represents the sequence length and each row contains the 4D vector representing the respective nucleotide. This representation facilitates efficient computational processing of DNA sequences in neural network architectures while preserving the discrete categorical nature of nucleotide sequences essential for accurate feature extraction and subsequent model training.

### Model architecture

The ETNet architecture comprises three principal components: (i) a CNN module for local feature extraction, (ii) a Transformer module for global sequence information processing, and (iii) a prediction head for interaction classification (Fig. 1). The model processes two one-hot encoded DNA sequences, each 2000 bp in length, to predict EEIs in the human genome. Initially, convolutional blocks with pooling layers systematically reduce the spatial dimension from 2000 to 500 bp, resulting in position vectors where each represents 128 bp of genomic context. A single-layer multilayer perceptron (MLP) subsequently performs nonlinear transformation on these extracted local features, enhancing their representational capacity. The transformed features are then processed by a Transformer module that efficiently captures long-range global interactions between the sequence representations through its self-attention mechanisms. Finally, two prediction heads determine the likelihood of EEIs by integrating information from both processed enhancer sequences. ETNet incorporates complete Transformer encoder blocks [27], each comprising multi-head self-attention, position-wise feed-forward networks, and layer normalization, rather than single-layer attention mechanisms used in conventional genomic models. This architecture enables parallel attention computation across multiple representation subspaces, allowing simultaneous capture of diverse enhancer features including motif composition, sequential dependencies, and regulatory context. The multi-head attention mechanism effectively models complex long-range dependencies characterizing functional genomic interactions, while the complete encoder structure provides a robust framework for transfer learning across cellular contexts. The detailed architecture and hyperparameter configuration are presented in Fig. 1 and Supplementary Table 2.

### Key technical components

#### Local feature extraction via convolutional neural network

The CNN module employs 128 convolutional kernels of size 9 × 9, strategically designed to correspond to the characteristic scale of DNA motifs—critical regulatory elements in genomic sequences. This architectural configuration enables efficient identification of functional regions within enhancer DNA sequences. When scanning regions containing regulatory elements, the convolutional operations generate feature vectors with distinctive activation patterns, substantially different from those produced from nonfunctional regions. This differentiation in signal patterns provides essential discriminative information for downstream analysis. ETNet maximizes the utility of these features through the subsequent Transformer architecture, where the self-attention mechanism automatically assigns higher weights to vectors containing significant biological signals, thereby prioritizing functionally relevant genomic regions.

#### Global feature extraction via transformer

Building upon the locally extracted features, the Transformer module employs a Multi-head Attention Mechanism (MHAM) to capture long-range interactions between enhancer sequences. The MHAM architecture comprises eight attention heads, each processing information with a value dimension of 1024 and a key/query dimension of 1000. Each attention head independently transforms input sequences into three components: queries (representing current positional information), keys (information to be attended to), and values (information to be propagated). The attention matrix is computed through the dot product of queries and keys, incorporating relative positional encoding to maintain spatial context within the genomic sequence. Prior to the MHAM layer, an MLP transforms the nucleotide segments into a rich nonlinear representation space. This transformation enhances the model’s ability to capture the cooperative functionality of adjacent nucleotides within regulatory motifs. Through this hierarchical processing approach—from local feature extraction to global information integration—ETNet effectively identifies and characterizes long-range chromatin interactions while maintaining interpretability of the underlying regulatory mechanisms that govern EEIs.

### Training and evaluation

#### Training details

ETNet was trained on an NVIDIA A800 graphics processing unit (GPU) using the Adam optimizer with an initial learning rate of 0.00001 for 50 epochs and a batch size of 100. For cross-cell line transfer learning experiments, we implemented a fine-tuning protocol with a reduced learning rate of 0.0001 to facilitate effective knowledge transfer while preserving previously learned representations.

#### Evaluation metrics

Model performance was assessed using multiple complementary metrics: AUC, Accuracy (ACC), F1-score, Precision, and Area Under the Precision–Recall Curve (AUPR). These metrics provide a comprehensive evaluation framework that addresses various aspects of model performance in the context of chromatin interaction prediction. The fundamental binary classification metrics are defined as:


$$ Accuracy=\frac{TP+ TN}{TP+ TN+ FP+ FN} $$

$$ Precision=\frac{TP}{TP+ FP} $$

$$ Recall=\frac{TP}{TP+ FN} $$

$$ F1=2\cdotp \frac{Precision\times Recall}{Precision+ Recall} $$

where TP (true positives) represents correctly predicted chromatin interactions, TN (true negatives) represents correctly identified noninteracting regions, FP (false positives) represents incorrectly predicted interactions, and FN (false negatives) represents missed interaction predictions.

The receiver operating characteristic (ROC) curve is constructed by plotting the true positive rate (TPR) against the false positive rate (FPR) at various classification thresholds:


$$ FPR=\frac{FP}{FP+ TN} $$

$$ TPR=\frac{TP}{TP+ FN} $$

To address class imbalance inherent in chromatin interaction datasets, we additionally employed the area under the precision–recall curve (AUPR):


$$ AUPR=\int Precision(Recall), dRecall $$

For fair comparison across methodologies, all evaluated models were executed with consistent hyperparameter configurations where applicable, and input data were preprocessed according to each model’s specific requirements while maintaining information integrity across comparative analyses.

#### Deep learning important feature analysis

To quantify the contribution of individual nucleotides to chromatin interaction predictions, we implemented Deep Learning Important Features (DeepLIFT), an advanced interpretability methodology that assigns importance scores based on feature contributions to specific model outputs. DeepLIFT calculates contribution scores by systematically comparing input sequences with reference sequences generated through dinucleotide shuffling. This computational shuffling approach preserves local sequence composition while effectively disrupting potential regulatory motifs, thereby creating appropriate baseline references. The quantitative differences in model predictions between original input sequences and their shuffled references are subsequently utilized to determine the impact of specific sequence features. This comprehensive analysis facilitates identification of the most influential DNA sequence elements in chromatin interaction predictions, thereby providing mechanistic insights into the sequence basis of long-range regulatory relationships within the genome [28–31].

#### Motif identification and enrichment analysis

To identify transcription factor binding motifs from learned sequence features, we employed a sliding-window motif matching approach. Nucleotide attribution scores generated by DeepLIFT were converted to position weight matrix format and compared against human transcription factor motifs from the JASPAR database. The average length of all motifs was used as the sliding window size to identify similar regulatory elements. Motif similarity was evaluated using cosine similarity, with a threshold of 0.8 applied to select the best JASPAR match for each predicted motif. Matching results were saved in MEME format and included frequency counts for each motif, facilitating identification of cell-type-specific enrichment patterns across GM12878, K562, and MCF-7 cell lines.

#### Higher-order enhancer–enhancer interaction analysis using greedy search

We developed a greedy search–based computational approach to systematically identify and quantify functional interactions between key regulatory regions in EEIs. Through iterative perturbation analysis, the methodology progressively identifies region combinations with maximal influence on prediction outcomes. The analytical process begins by dividing test sequences into nonoverlapping regions, utilizing the original sequence as a baseline. In each iteration, candidate regions undergo perturbation testing through multiple dinucleotide shuffling events to minimize stochastic effects [32, 33], with perturbation impacts on model predictions being systematically recorded. The most critical region is selected based on predefined optimization objectives (maximizing or minimizing prediction changes), its perturbation is permanently retained, and the region is subsequently removed from the candidate pool. This process continues with updated sequences until reaching the specified iteration limit or exhausting all regions. For each iteration, we document baseline prediction values, prediction change distributions following perturbations, selected critical regions with their precise genomic locations, and average predictive effects. This comprehensive data enables construction of regional importance hierarchies and quantification of inter-region dependencies. The methodology’s key advantages include context-dependent analysis through selection based on previously perturbed regions, capture of nonlinear higher-order interactions, enhanced measurement stability through multiple randomization and result averaging, and reduced computational complexity via greedy strategy, enabling large-scale sequence analysis. The resulting regional importance rankings and interaction patterns provide crucial experimental evidence and theoretical guidance for understanding complex gene regulatory networks and designing functional DNA sequences [18].

#### Transfer learning fine-tuning strategy

To evaluate cross-cell type transfer learning capability, we implemented a selective fine-tuning strategy that adapts the input layer, convolutional layers, and output layer while keeping intermediate Transformer layers frozen. This approach preserves learned cell-type-invariant features while allowing adaptation to cell-type-specific patterns. Fine-tuning was performed using the Adam optimizer with a learning rate of 0.0001 for 30 epochs with early stopping patience of 5. This strategy leverages complementary architectural strengths: convolutional layers adapt to scan cell-type-specific local motif patterns, while frozen Transformer layers retain generalizable sequence features learned during initial training. Selective layer adaptation enables efficient knowledge transfer across cellular contexts with minimal parameter updates.

#### Baseline model training and evaluation

To ensure fair comparison, all baseline methods were trained and evaluated on our EEI datasets using identical experimental protocols. The baseline methods include traditional machine learning approaches (K-Nearest Neighbors, Gradient Boosting Machine, XGBoost, Logistic Regression), deep learning methods originally designed for chromatin interaction or EPI prediction (lollipop, deepTACT, EPInformer, EPI_DLMH, deepPHiC), and the EEI-specific method EnContact. All methods used the same dataset partitioning (8:1:1 train/validation/test split) and one-hot sequence encoding. For deep learning methods, consistent training hyperparameters were applied including Adam optimizer, learning rate 1e-5, and early stopping with patience of 5 epochs. For methods originally designed for EPI prediction, only the input layer was modified to accommodate enhancer–enhancer sequence pairs while all other architectural components and training procedures were preserved. Complete implementation code for all baseline models has been made publicly available in the GitHub repository.

#### DeLong method

The DeLong method [34] is a nonparametric statistical approach used to estimate the variance of the AUC and to perform significance comparisons between AUCs of different models. Based on U-statistics, it quantifies a model’s ability to discriminate between positive and negative samples by comparing each positive sample against all negative samples. This method can provide confidence intervals for a single model’s AUC and also assess whether the difference between AUCs of two related or independent models is statistically significant, thereby evaluating the reliability and robustness of model performance.

## Results

### Optimization of convolutional neural network architecture and sequence standardization strategy

**Figure 2:**
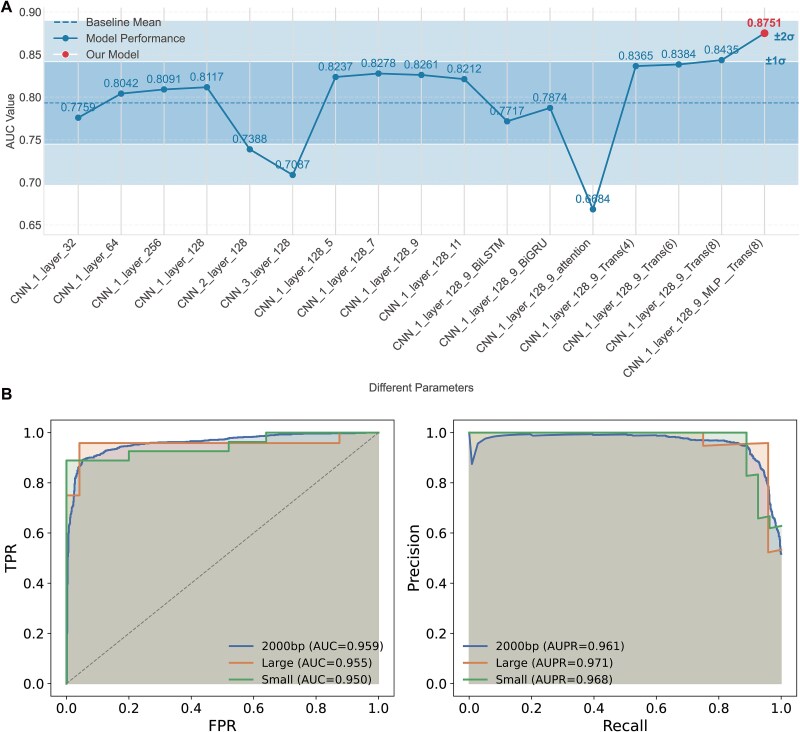
CNN architecture optimization and sequence length validation. (A) Performance evaluation across architectural configurations measured by AUC, with shaded regions representing ±1σ and ± 2σ. Horizontal dashed line indicates baseline performance; rightmost point highlights optimized ETNet configuration. (B) Performance comparison across enhancer size categories (small ≤541 bp, large >541 bp, and standardized 2000 bp) demonstrating robust prediction independent of native enhancer length.

We systematically optimized the CNN architecture and sequence standardization strategy for effective DNA feature extraction. DNA sequences exhibit linear sequential patterns analogous to natural language text, making them amenable to processing through 1D CNNs. The sliding window mechanism of CNNs effectively captures biologically significant local patterns, particularly transcription factor binding motifs. We evaluated the impact of convolutional layer depth on predictive performance and found that increasing network depth correlated with declining AUC, contrary to conventional deep learning principles. This performance degradation resulted from amplified noise signals in deeper networks, which exhibited heightened sensitivity to biological noise that obscured regulatory features. We therefore adopted a single-layer convolutional architecture with optimized kernel dimensions to precisely capture essential biological features while minimizing noise interference (Fig. 2A, Supplementary Fig. 1).

To ensure robust feature extraction across enhancers of varying native sizes, we validated our 2000 bp sequence standardization strategy through size-stratified evaluation in K562 cells. Analysis of the FANTOM5 dataset showed that 95% of enhancers are ≤541 bp, yet substantial size heterogeneity exists. We categorized enhancers into large (>541 bp, *n* ≈ 500) and small-to-medium (≤541 bp) groups with balanced subsampling to eliminate size bias. EEI pairs were annotated using ChIA-PET data for each category. Performance evaluation demonstrated minimal size-dependent variation, with AUC values of 0.959 (mixed), 0.955 (large), and 0.950 (small) and AUPRC values of 0.961 (mixed), 0.971 (large), and 0.968 (small). Maximum differences were 0.009 for AUC and 0.010 for AUPRC (Fig. 2B), confirming that the 2000 bp window enables consistent feature extraction across the enhancer size spectrum regardless of native enhancer length.

### Transformer-based global feature integration improves long-range dependency Modeling

For global feature extraction, we conducted rigorous comparative experiments evaluating four sequence modeling architectures: Bidirectional Long Short-Term Memory (BLSTM), Bidirectional Gated Recurrent Unit (BGRU), classical attention mechanism, and Transformer. Each architecture offers distinct advantages for capturing long-range dependencies within DNA sequences, a crucial capability for accurate chromatin interaction prediction. Using identical datasets and standardized evaluation protocols, our systematic assessment demonstrated the Transformer architecture’s superior capability in modeling long-range dependency features within enhancer DNA sequences. This finding aligns with the Transformer’s established effectiveness in natural language processing domains, validating its transferability to genomic sequence analysis for regulatory interaction prediction.

**Figure 3:**
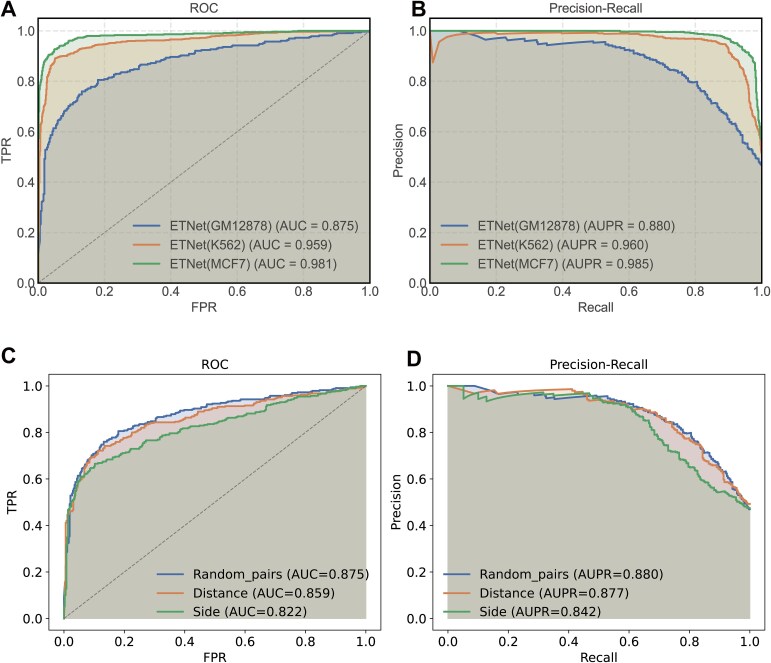
ETNet performance evaluation and negative sampling strategy comparison. (A, B) Cell-type-specific performance across GM12878, K562, and MCF-7 measured by ROC and precision-recall curves, demonstrating consistent predictive capability. (C, D) performance comparison of three negative sampling strategies in GM12878, showing minimal difference between random pairing and distance-based sampling.

The Transformer’s self-attention mechanism enables direct relationship establishment between elements at arbitrary positions within the sequence, facilitating identification of functionally related patterns distributed throughout enhancer regions. After establishing the Transformer architecture’s superiority, we optimized its core component—the multi-head attention mechanism. Through systematic evaluation of various head configurations (4, 6, and 8), we determined that an 8-head attention structure achieved optimal balance between feature expression richness and computational efficiency. This configuration provides sufficient representational capacity for identifying complex chromatin interaction patterns while maintaining efficient training and inference performance (Fig. 2A). Throughout the parameter optimization process, we employed five distinct metrics (AUC, ACC, F1-score, Precision, and AUPR) for comprehensive performance assessment across all architectural variants (Supplementary Fig. 1). ETNet demonstrated strong predictive performance across GM12878, K562, and MCF-7 cell lines, with performance metrics in the following ranges: accuracy (0.8146–0.9351), F1 score (0.7936–0.9338), and AUC (0.8751–0.9815). Peak performance was observed in the MCF-7 cell line (Fig. 3A and B, Supplementary Fig. 2). These consistent results across diverse cell types validate ETNet’s effectiveness and adaptability for EEI prediction in varied cellular contexts.

To evaluate potential bias from negative sample generation, we systematically compared three strategies: distance-based sampling matching the genomic distance distribution of positive pairs, fixed-end shuffling preserving one enhancer while randomizing the partner, and completely random pairing. Evaluation in GM12878 demonstrated that distance-based sampling achieved performance highly similar to random pairing, with the AUC differing by only 0.016 and the AUPRC by 0.003 (Fig. 3C and D). The fixed-end strategy showed larger performance differences, likely reflecting reduced sample diversity. These results indicate that random pairing does not substantially inflate performance estimates, as the biologically motivated distance-matched strategy yields comparable results. Based on these findings and to maintain sample diversity while ensuring stable model training, we selected random pairing as the primary negative sample generation strategy.

### Comprehensive comparative analysis validates model superiority

To rigorously evaluate ETNet, we established a comprehensive comparative experimental framework against both specialized and related methodologies. Our primary benchmark comparison was conducted against EnContact, the current state-of-the-art method in EEI prediction. Given the limited availability of specialized methods in this domain, we expanded our analysis to include high-performing approaches from EPI prediction, specifically incorporating deepTACT, deepPHiC, EPI_DLMH, lollipop, and the recently published EPInformer. For comprehensive evaluation, we also included classical machine learning algorithms as baseline references (K-Nearest Neighbors, XGBoost, Gradient Boosting Machines, and Logistic Regression). To maintain experimental rigor, we implemented critical control measures across three cell lines (GM12878, K562, and MCF-7), employing identical data preprocessing pipelines, feature encoding strategies, and standardized hyperparameters including training iterations, batch size, and learning rate. Evaluation metrics and testing procedures were kept consistent across all comparisons, ensuring performance differences genuinely reflected algorithmic advantages rather than experimental variations.

**Figure 4:**
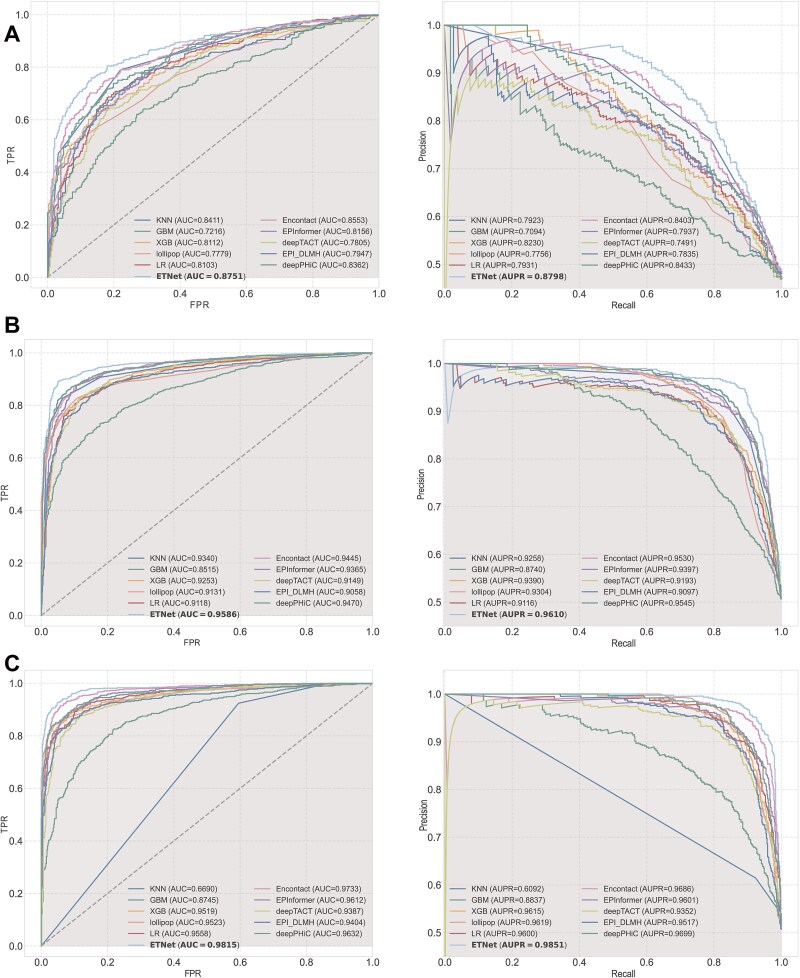
Comprehensive baseline comparison across cell lines. Systematic evaluation of ETNet against established methods including EnContact, deepPHiC, EPInformer, and classical machine learning approaches across GM12878, K562, and MCF-7, quantified through ROC and precision–recall metrics.

Experimental results demonstrated that ETNet achieved superior performance across all three cell lines (Fig. 4, Supplementary Fig. 3, Supplementary Table 7). For GM12878, ETNet achieved an AUC of 0.875 and an AUPR of 0.880; for K562, an AUC of 0.959 and an AUPR of 0.961; and for MCF-7, an AUC of 0.982 and an AUPR of 0.985. These results consistently surpassed all baseline methods across all evaluation metrics. Among baseline methods, performance patterns varied across cell types. In GM12878, EnContact demonstrated stronger performance in AUC (0.855), accuracy (0.778), and F1 score (0.769) compared to deepPHiC (AUC 0.836, accuracy 0.755, F1 0.746), though both methods achieved comparable AUPR. In K562, deepPHiC showed advantages across metrics (AUC 0.947, AUPR 0.955, accuracy 0.885, F1 0.887) over EnContact (AUC 0.945, AUPR 0.953, accuracy 0.871, F1 0.863). In MCF-7, EnContact achieved higher AUC (0.973), accuracy (0.915), and F1 score (0.911) compared to deepPHiC (AUC 0.963, accuracy 0.901, F1 0.901), with comparable AUPR values. These cell-type-specific performance patterns indicate that both EnContact and deepPHiC achieve strong baseline performance with context-dependent advantages. ETNet’s consistent superiority across all cell lines and metrics—with improvements of ~3%–5% in both AUC and F1 scores relative to the best-performing baseline in each cell type—demonstrates the effectiveness of integrating convolutional feature extraction with Transformer-based global dependency modeling for EEI prediction. The comprehensive nature of our comparative analysis framework, incorporating both specialized and related methodologies with standardized experimental protocols, provides robust validation of ETNet’s predictive capabilities for EEIs across diverse cellular contexts.

### Cross-cell-type enhancer-enhancer interaction prediction through transfer learning

**Figure 5:**
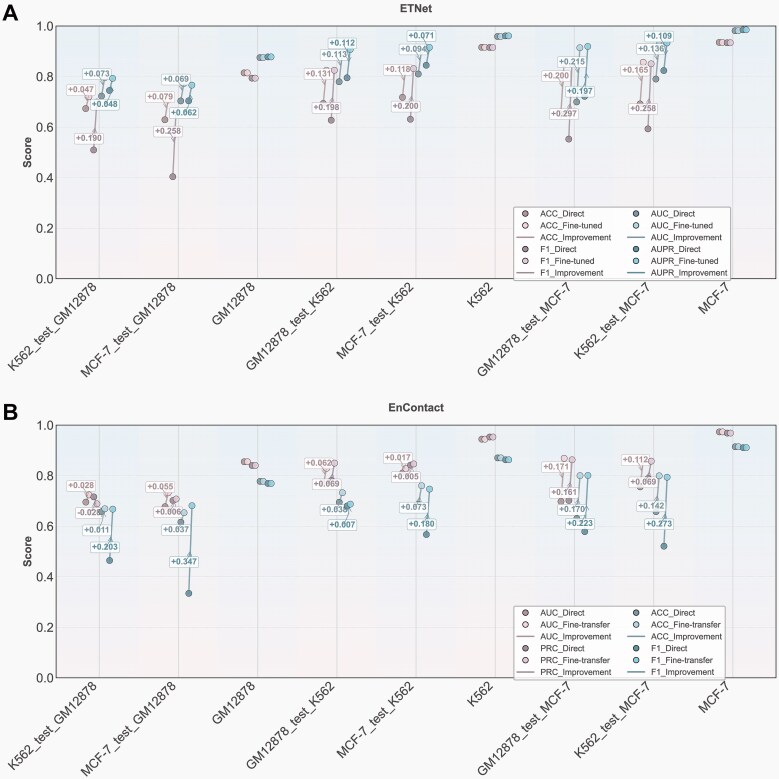
Cross-cell-type transfer learning performance. (A) ETNet performance comparison between direct application and selective fine-tuning across all cell line pairs, demonstrating substantial improvements through minimal parameter adaptation. (B) EnContact performance under identical transfer learning protocols, showing ETNet’s architectural advantages for cross-cell-type generalization.

We conducted comprehensive cross-cell type validation across all pairwise combinations of GM12878, K562, and MCF-7 cell lines to evaluate ETNet’s generalization capability. To address the challenge of cell-type-specific enhancer activity patterns, we implemented a selective fine-tuning strategy that exclusively adapts the input and output layers while preserving the intermediate feature extraction layers. This approach leverages the observation that core DNA sequence features captured during initial training remain largely cell-type-invariant, enabling efficient knowledge transfer through minimal parameter adaptation. Direct cross-cell type prediction without fine-tuning yielded moderate performance, with AUC values ranging from 0.700 to 0.810. However, selective fine-tuning substantially improved predictive accuracy across all cell line combinations, achieving AUC values of 0.773–0.927. The most pronounced improvements were observed for transfers from GM12878 to MCF-7 (AUC: 0.700 → 0.914, +0.215) and K562 to MCF-7 (AUC: 0.790 → 0.927, +0.137), with similar substantial gains across all evaluation metrics (Fig. 5A, Supplementary Fig. 4A).

To assess the architectural advantages of the Transformer framework for transfer learning, we performed identical cross-cell type experiments using EnContact, a conventional attention-based model. ETNet consistently outperformed EnContact in both direct prediction and fine-tuning scenarios across all tested combinations. For example, transferring from K562 to MCF-7 achieved AUCs of 0.927 versus 0.867, while transferring from MCF-7 to K562 yielded AUCs of 0.904 versus 0.828 for ETNet and EnContact, respectively (Fig. 5B, Supplementary Fig. 4B). These results demonstrate that the complete Transformer encoder architecture, with its multi-head attention mechanism and hierarchical feature processing, enables superior learning of cell-type-invariant sequence features compared to conventional single-layer attention mechanisms. This robust cross-cell type generalization capability addresses a critical limitation in current chromatin interaction prediction methods, enabling accurate EEI prediction in poorly characterized cell types by leveraging knowledge from well-annotated cell lines through minimal fine-tuning.

### Transfer learning enables cross-task enhancer–promoter interaction prediction

To evaluate whether ETNet captures generalizable principles of chromatin interactions beyond enhancer–enhancer specificity, we conducted cross-task transfer experiments from EEI to EPI prediction. We constructed a balanced EPI dataset from TargetFinder encompassing GM12878 and K562 cell lines (Supplementary Table 3). This experimental design tests whether sequence-encoded regulatory features and long-range dependency representations learned from EEI data can transfer to EPI prediction, motivated by shared biological mechanisms including chromatin looping, Mediator complex recruitment, Cohesin-mediated contacts, and CTCF-mediated insulation. We employed selective fine-tuning where intermediate layers remain frozen while only input and output layers are updated, reducing computational requirements while preserving learned feature extraction capabilities.

The model achieved robust EPI prediction performance: AUC of 0.861, F1 score of 0.793, and accuracy of 0.792 for GM12878 and AUC of 0.897, F1 score of 0.855, and accuracy of 0.858 for K562 (Supplementary Table 4). These results demonstrate that ETNet learns general principles of chromatin interaction rather than EEI-specific patterns, enabling knowledge transfer across interaction types. This cross-task transferability has practical implications for cell types with limited EPI training data, where EEI data can serve as pretraining to reduce labeled data requirements. We emphasize that while this validates shared regulatory mechanisms, EEI and EPI prediction remain distinct tasks and ETNet trained on EEI data does not replace dedicated EPI models.

### Integrated feature attribution reveals critical motifs and nonlinear cooperative mechanisms between enhancers

**Figure 6:**
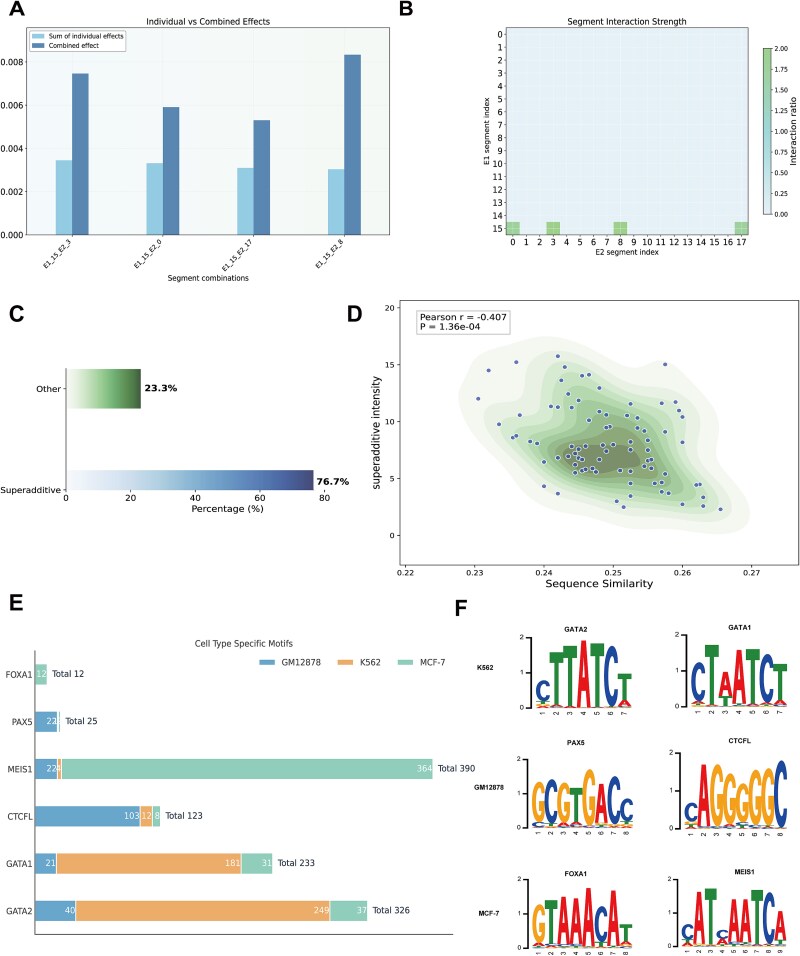
Feature attribution analysis reveals cooperative mechanisms and cell-type-specific motifs. (A) Comparison of summed individual effects versus combined synergistic effects for example enhancer pair chr22:46995378–46997378 and chr8:129203880–129205880; (B) heatmap identifying coordinated high-importance regions across both enhancers; (C) distribution of super-additive effects across all enhancer pairs in GM12878 (76.7% super-additive, 23.3% other); (D) negative correlation between super-additivity strength and sequence similarity (*r* = −0.407, *P* = 1.36 × 10^−4^); (E) cell-type-specific transcription factor motif enrichment showing GATA2 predominance in K562, PAX5 in GM12878, and FOXA1 in MCF-7; and (F) sequence logos of representative lineage-specific motifs.

To validate sequence feature learning and investigate enhancer cooperative mechanisms, we employed DeepLIFT for attribution analysis and developed a greedy search algorithm to identify functionally important regions through dinucleotide shuffling perturbations. We compared the summed importance scores of individual enhancer regions with their synergistic importance scores under cooperative conditions (Fig. 6A) and identified regions exhibiting simultaneously high importance in both interacting enhancers (Fig. 6B). To systematically characterize cooperative mechanisms across the genome, we performed statistical analysis of synergistic effects for all enhancer interaction pairs in the GM12878 test dataset. Results showed that 76.7% of enhancer pairs exhibited super-additivity, while 23.3% displayed additive or sub-additive patterns (Fig. 6C), indicating that cooperative interactions represent a prevalent regulatory mode. We further examined whether super-additivity strength correlates with sequence properties of interacting enhancers. Analysis revealed a significant negative correlation between super-additivity strength and sequence similarity (*r* = −0.407, *P* = 1.36 × 10^−4^, Fig. 6D), suggesting that enhancers with greater sequence divergence tend to exhibit stronger cooperative effects. This finding is consistent with a functional complementarity model where distinct enhancers contribute different regulatory inputs to achieve synergistic transcriptional control, providing computational evidence for complex nonlinear regulatory mechanisms underlying enhancer interactions.

To assess whether ETNet captures biologically relevant cell-type-specific regulatory features, we performed systematic motif enrichment analysis across GM12878, K562, and MCF-7 cell lines. DeepLIFT attribution scores were converted to position weight matrix format and compared with human transcription factor motifs from the JASPAR database. Motif scanning revealed distinct lineage-specific enrichment patterns consistent with known transcription factor activities. GATA2 motifs showed pronounced enrichment in K562 (249 occurrences) compared to GM12878 (40 occurrences) and MCF-7 (37 occurrences), consistent with the established role of GATA-binding protein (GATA) factors in myeloid and erythroid lineage regulation. PAX5 motifs exhibited preferential enrichment in GM12878 (22 occurrences) versus K562 (1 occurrence) and MCF-7 (2 occurrences), reflecting its function as a master regulator of B-cell development. FOXA1 motifs appeared exclusively in MCF-7 (12 occurrences) with no detection in hematopoietic cell lines, aligning with its role as a pioneer factor in epithelial and breast cancer cell regulation (Fig. 6E and F). These cell-type-specific motif enrichment patterns align with extensive experimental evidence from ChIP-seq and functional studies, demonstrating that ETNet effectively learns lineage-specific regulatory features underlying EEIs and recovers known cell-selective enhancer binding patterns from the literature.

### Computational analysis of single-nucleotide polymorphism effects on predicted enhancer–enhancer interactions in JAK–STAT pathway genes

Previous studies identified six SNPs (rs560898780, rs1257658099, rs370669851, rs571421696, rs910130021, and rs138606888) located within enhancer regions of genes involved in the JAK–STAT pathway (IRF3, IL7R, JAK2, JAK3, SOCS1, and PTPN2), with potential effects on gamma-interferon-activated site (GAS) motif structure (Supplementary Table 5) [35]. Transcriptomic analysis revealed differential expression in JAK2, SOCS1, and PTPN2 genes under various stimulatory conditions (IFNα, IFNβ, IFNγ, IL-6, and IL-7), suggesting potential relationships between GAS motif alterations and JAK–STAT pathway activity.

**Figure 7:**
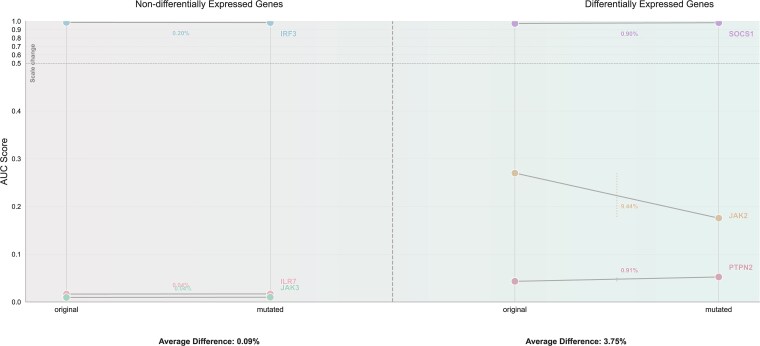
Computational analysis of SNP effects on predicted enhancer interactions in JAK–STAT pathway genes. Differential prediction changes between reference and variant alleles for six SNPs in enhancer regions of IRF3, IL7R, JAK2, JAK3, SOCS1, and PTPN2. Nondifferentially expressed genes (left) show minimal prediction changes (0.09% average), while differentially expressed genes under cytokine stimulation (right) exhibit larger changes (3.75% average), with JAK2 showing the most pronounced effect (9.44%).

To explore whether these SNPs might influence predicted EEIs through motif alterations, we conducted computational analysis using ETNet. We selected 150 bp sequences upstream of each SNP locus as anchor1 and combined them with corresponding chromosomal enhancers from our dataset as anchor2. Comparison of predicted interaction probabilities between reference and variant alleles revealed differential responses: variants in JAK2, SOCS1, and PTPN2 genes showed average prediction value changes of 3.75%, while variants in IRF3, IL7R, and JAK3 genes exhibited minimal changes of 0.09% (Fig. 7). This computational pattern shows correspondence with the expression data from the previous study, suggesting that these SNPs may represent candidate variants for experimental investigation.

We emphasize that these findings are entirely computational and exploratory in nature. ETNet predicts interaction probability rather than providing mechanistic explanation of how SNPs affect regulatory function. The observed prediction changes represent model-inferred effects based on sequence alterations, not experimentally validated regulatory outcomes. These results should be interpreted as hypothesis-generating, identifying candidate variants that may influence enhancer cooperation in JAK–STAT pathway regulation and warrant experimental validation through approaches such as reporter assays, CRISPR-based enhancer perturbation, or allele-specific ChIP-seq analysis.

### ETNet performance in capturing super-enhancer regulatory architecture

**Figure 8:**
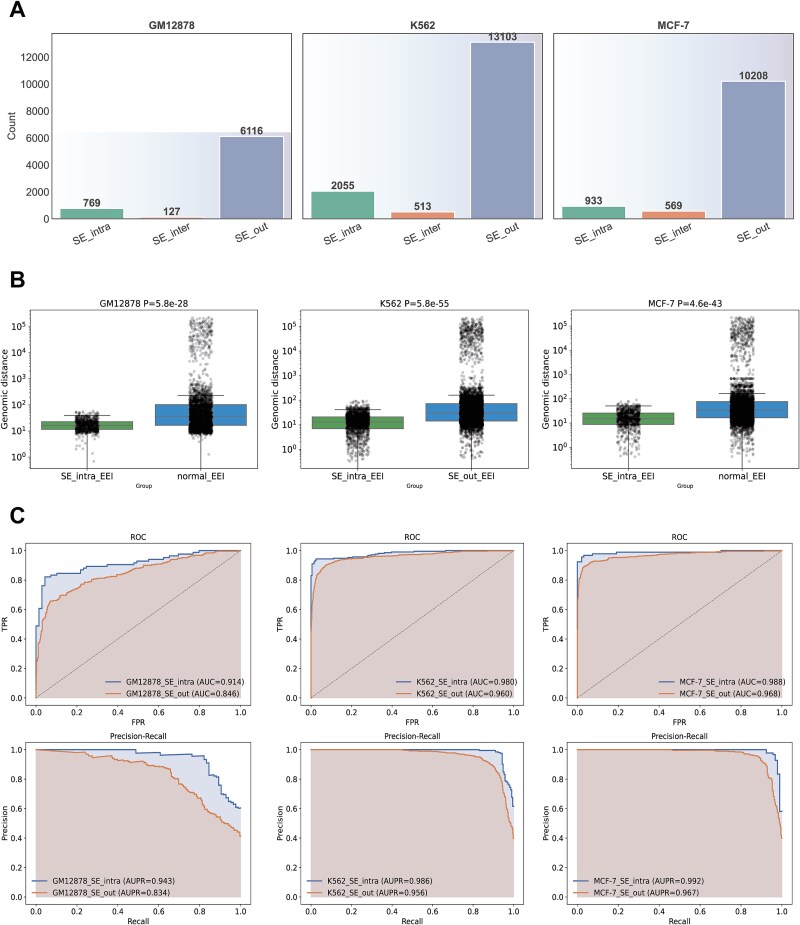
ETNet performance on super-enhancer regulatory architecture. (A) Distribution of enhancer–enhancer interactions within super-enhancers (SE_intra), between super-enhancers (SE_inter), and outside super-enhancers (SE_out) across GM12878, K562, and MCF-7; (B) genomic distance comparison showing SE_intra interactions are significantly shorter than SE_out interactions (all *P* < 10^−20^); and (C) performance evaluation demonstrating higher accuracy for SE_intra compared to SE_out interactions across all cell lines.

To evaluate ETNet’s capability to handle the complexity of super-enhancer regulatory architecture, we performed a comprehensive analysis using super-enhancer annotations from the dbSUPER database. All EEIs were systematically categorized into three types: SE_intra (both enhancers within the same super-enhancer), SE_inter (enhancers in different super-enhancers), and SE_out (interactions not associated with any super-enhancer). Analysis revealed cell-type-specific distributions, with SE_intra representing 769 (11.2%), 2055 (13.2%), and 933 (8.3%) of total interactions in GM12878, K562, and MCF-7, respectively, while the majority of interactions fall into the SE_out category (Fig. 8A). Genomic distance comparison demonstrated that SE_intra interactions are significantly shorter than SE_out interactions across all cell lines (*P* = 5.8 × 10^−28^ for GM12878, *P* = 5.8 × 10^−55^ for K562, *P* = 4.6 × 10^−43^ for MCF-7, Fig. 8B), consistent with the compact spatial organization characteristic of super-enhancers.

Performance evaluation demonstrated differential accuracy across interaction categories. ETNet achieved substantially higher predictive accuracy for SE_intra interactions compared to SE_out interactions across all three cell lines (Fig. 8C). In GM12878, SE_intra interactions achieved an AUC of 0.914 and an AUPRC of 0.943 versus an AUC of 0.846 and an AUPRC of 0.834 for SE_out interactions. Similar performance advantages were observed in K562 (AUC 0.980 versus 0.960, AUPRC 0.986 versus 0.956) and MCF-7 (AUC 0.988 versus 0.968, AUPRC 0.992 versus 0.967), indicating that the model effectively captures super-enhancer-specific sequence and regulatory features. Super-additivity analysis revealed that SE_intra interactions exhibit markedly stronger super-additive effects than SE_out interactions with clear cell-type specificity: 79.0% versus 36.8% in GM12878, 69.7% versus 11.1% in K562, and 61.2% versus 24.7% in MCF-7 (Supplementary Fig. 5A–F), supporting the model whereby enhancer clustering within super-enhancers drives synergistic transcriptional activation. Analysis of attention weights from the Transformer module uncovered hierarchical spatial organization, with ~24.8% of interactions receiving consistently higher attention weights, suggesting the existence of dominant regulatory hubs within super-enhancers (Supplementary Fig. 5G). Quantitative comparison showed that attention weight correlations between enhancer pairs within SE_intra interactions (*r* = 0.3847, *P* < 0.001) are significantly higher than random enhancer pairs (*r* = 0.0681, *P* = 0.403), reflecting coordinated regulatory function (Supplementary Fig. 5H and I). These findings demonstrate that ETNet not only identifies functional enhancer interactions within super-enhancers but also captures their hierarchical and cooperative spatial organization through the attention mechanism, revealing higher-order regulatory architecture.

### Comprehensive validation using cross-validation, stringent data partitioning, and DeLong statistical testing

To evaluate robustness against potential information leakage, we conducted two complementary validation strategies across GM12878, K562, and MCF-7 cell lines. First, 10-fold random cross-validation demonstrated highly consistent performance with the original 8:1:1 split. In GM12878, the mean AUC was 0.872 compared to the original 0.875, and the mean AUPRC was 0.890 compared to 0.880 (Supplementary Fig. 6A and B). In K562, the mean AUC was 0.960 versus 0.959 originally, and the mean AUPRC was 0.965 versus 0.961 (Supplementary Fig. 6C and D). In MCF-7, the mean AUC was 0.978 versus 0.981 originally, and the mean AUPRC was 0.981 versus 0.985 (Supplementary Fig. 6E and F). The minimal variation across folds indicates stable generalization performance independent of data partitioning.

To directly address potential information leakage from enhancer overlap between training and test sets, we implemented a dual-constraint enhancer-level splitting strategy. This approach ensures complete separation where any enhancer appearing in the test set is excluded from all training set interaction pairs, and each enhancer appears at most once in the test set through deduplication. This stringent partitioning reduced test set sizes from 1404 to 1159 in GM12878, 3134 to 2199 in K562, and 2342 to 1595 in MCF-7 (Supplementary Table 6). Under enhancer-level splitting, performance showed moderate decreases: in GM12878, the AUC decreased from 0.875 to 0.826 and the AUPRC from 0.880 to 0.821 (Supplementary Fig. 7A and B); in K562, the AUC decreased from 0.959 to 0.942 and the AUPRC from 0.961 to 0.926 (Supplementary Fig. 7C and D); in MCF-7, the AUC decreased from 0.982 to 0.967 and the AUPRC from 0.985 to 0.940 (Supplementary Fig. 7E and F). While performance declined under the strictest evaluation, the reductions remained modest, particularly in K562 and MCF-7 where larger training sets support robust generalization. These results demonstrate that ETNet maintains strong predictive capability even when evaluated under conditions designed to completely eliminate potential information leakage.

To assess the statistical significance of ETNet’s performance improvement over EnContact, we conducted DeLong tests across six cell lines including the initial three (GM12878, K562, MCF-7) and three additional cell lines (IMR90, HCT116, HCASMC). Results demonstrated that ETNet achieved significantly higher AUC values than EnContact across all cell lines: GM12878 (AUC 0.860 versus 0.805, *P* = .013157), K562 (AUC 0.953 versus 0.932, *P* = .013525), MCF-7 (AUC 0.978 versus 0.965, *P* = .045820), IMR90 (AUC 0.913 versus 0.889, *P* = .017035), HCT116 (AUC 0.953 versus 0.935, *P* = .004461), and HCASMC (AUC 0.814 versus 0.785, *P* < .001) (Supplementary Fig. 8). All *P*-values were below .05, indicating that ETNet’s performance improvements are statistically significant across diverse cellular contexts including hematopoietic, epithelial, mesenchymal, and vascular lineages.

## Discussion

Over the past decades, researchers have primarily focused on predicting EPIs, with relatively limited attention given to EEIs. The present study offers novel insights into EEI prediction and demonstrates that ETNet exhibits substantial competitive advantages compared to existing DNA sequence–based methods. ETNet maintains stable performance across different cell types with only minor adjustments to input and output layers during fine-tuning, suggesting that despite the cell-specific nature of enhancers, the integration of convolutional feature extraction and Transformer-based global dependency modeling effectively captures both lineage-specific and conserved regulatory features. This architecture enables efficient knowledge transfer across cellular contexts, as demonstrated by consistent performance improvements over conventional attention-based approaches in cross-cell type experiments. The model’s transferability extends to EPI tasks, indicating potential to identify conserved regulatory mechanisms underlying various types of chromatin interactions, though we emphasize that EEI and EPI prediction remain distinct problems requiring task-specific optimization.

Our computational findings provide insights into the organizational principles of enhancer cooperation with implications for regulatory network biology. Analysis of synergistic effects revealed that 76.7% of enhancer pairs exhibit super-additive cooperative behavior, where combined regulatory contributions exceed the sum of individual effects. Critically, we observed that super-additivity strength correlates negatively with sequence similarity between enhancer pairs (*r* = −0.407, *P* = 1.36 × 10^−4^), suggesting that sequence divergence and motif complementarity drive cooperative enhancement. This pattern is consistent with a functional complementarity hypothesis—where enhancers with distinct sequence features achieve stronger synergistic activation—which has important implications for how cells maintain transcriptional precision and robustness during developmental transitions. The prevalence of super-additive interactions indicates that cooperative enhancer regulation represents a widespread organizational principle governing gene expression programs. Furthermore, our cell-type-specific motif analysis demonstrates that enhancer interactions are governed by lineage-specific transcription factor repertoires, with GATA motifs predominating in hematopoietic cells, PAX5 in B lymphoid cells, and FOXA1 exclusively in epithelial cells [36–38]. These patterns illustrate how the same architectural framework—enhancer cooperation through chromatin looping—can be repurposed across developmental lineages through differential transcription factor utilization, enabling cell-type-specific transcriptional programs while maintaining common structural principles.

From a disease genetics perspective, disruption of EEIs may underlie various complex diseases. SNPs or epigenetic modifications may perturb the synergistic activity of specific EEIs, thereby affecting expression of key developmental or immune regulatory genes. Many risk variants reside within regulatory DNA regions, and structural variants can result in enhancer repositioning (“enhancer hijacking”) or disruption of insulating boundaries between regulatory domains, leading to pathogenic rearrangements [39]. Our computational analysis of SNPs in JAK–STAT pathway genes provides proof-of-concept for how EEI predictions might guide functional variant prioritization, though experimental validation remains essential. Disease phenotypes may often arise not from single enhancer malfunction but from cumulative effects of disrupted cooperative enhancer networks. Integrating EEI predictions from ETNet with enhancer–gene connectivity maps such as the Activity-by-Contact model could prioritize sets of cooperating enhancers that may jointly regulate disease-associated loci, enabling hypothesis generation for multi-perturbation experimental designs [40]. For instance, two variants in different cooperating enhancers may each exert modest individual effects but could induce synergistic regulatory changes when co-occurring, a scenario our super-additivity analysis suggests may be common.

However, our approach presents important limitations requiring acknowledgment. First, conclusions regarding super-additive effects and SNP impacts are based on computational attribution and model predictions rather than experimental validation. These findings should be interpreted as hypothesis-generating, identifying patterns and candidate variants that warrant experimental follow-up through approaches such as CRISPR perturbation, reporter assays, or allele-specific ChIP-seq. Second, while our motif analysis identifies transcription factor binding sites consistent with known regulatory factors, the functional roles of these motifs in mediating enhancer cooperation require experimental confirmation. Third, ETNet predicts interaction probability rather than providing a mechanistic interpretation of regulatory mechanisms, and predicted effects represent model inferences that must be validated experimentally to establish biological causality.

Future work should address several key directions. First, incorporating additional genomic features such as transcription factor binding profiles, chromatin accessibility data, and histone modification patterns may further enhance prediction accuracy and provide complementary regulatory information. Second, experimental investigation is needed to determine how cooperative effects between enhancers change when these enhancers simultaneously interact with shared target promoters and whether such multi-way interactions exhibit synergistic or antagonistic regulatory properties. Third, existing evidence demonstrates that EPIs exhibit dynamic transitions during development, with some interactions persisting throughout development while new interactions form progressively to activate developmental gene expression programs. Whether EEIs follow similar developmental dynamics, and how reorganization of enhancer cooperation networks contributes to cell fate determination and tissue-specific gene expression, represents a promising research direction that could illuminate fundamental principles of developmental gene regulation.

**Key Points**

- ETNet integrates convolutional neural networks with Transformer architecture to predict enhancer–enhancer (EEI) interactions from DNA sequences, achieving superior performance across multiple cell lines with statistical validation confirming significant improvements over existing methods.
- The model demonstrates effective cross-cell type transfer learning through selective fine-tuning strategies and shows transferability to enhancer–promoter interaction (EPI) prediction as exploratory investigation of shared chromatin interaction principles, though EEI and EPI prediction remain distinct tasks.
- Feature attribution analysis identified cell-type-specific regulatory motifs including GATA2, PAX5, and FOXA1, recovering lineage-specific transcription factor binding patterns consistent with experimental evidence and demonstrating that ETNet captures biologically relevant regulatory features.
- Computational analysis revealed that 76.7% of enhancer pairs exhibit super-additive cooperative behavior, with cooperativity strength negatively correlating with sequence similarity (*r* = −0.407, *P* = 1.36 × 10^−4^), providing computational evidence for functional complementarity in enhancer interactions that requires experimental validation.
- ETNet provides a framework for computational analysis of how genetic variations may influence regulatory networks, as demonstrated by proof-of-concept analysis of SNPs in JAK–STAT pathway genes, enabling hypothesis generation and variant prioritization for experimental investigation.

## Supplementary Material

Supplementary_Figure_1_bbaf634

Supplementary_Figure_2_bbaf634

Supplementary_Figure_3_bbaf634

Supplementary_Figure_4_bbaf634

Supplementary_Figure_5_bbaf634

Supplementary_Figure_6_bbaf634

Supplementary_Figure_7_bbaf634

Supplementary_Figure_8_bbaf634

Supplementary_Tables_bbaf634

## Data Availability

The source code for ETNet is open-sourced on GitHub at https://github.com/shuaibinw/ETNet.
