# Supplementary figures and images for: ETNet: an interpretable transformer framework for enhancer–enhancer interaction prediction with cross-context transferability
Source: Brief Bioinform. 2025 Nov 30;26(6):bbaf634. doi: 10.1093/bib/bbaf634 (PMC12665038; doi:10.1093/bib/bbaf634)

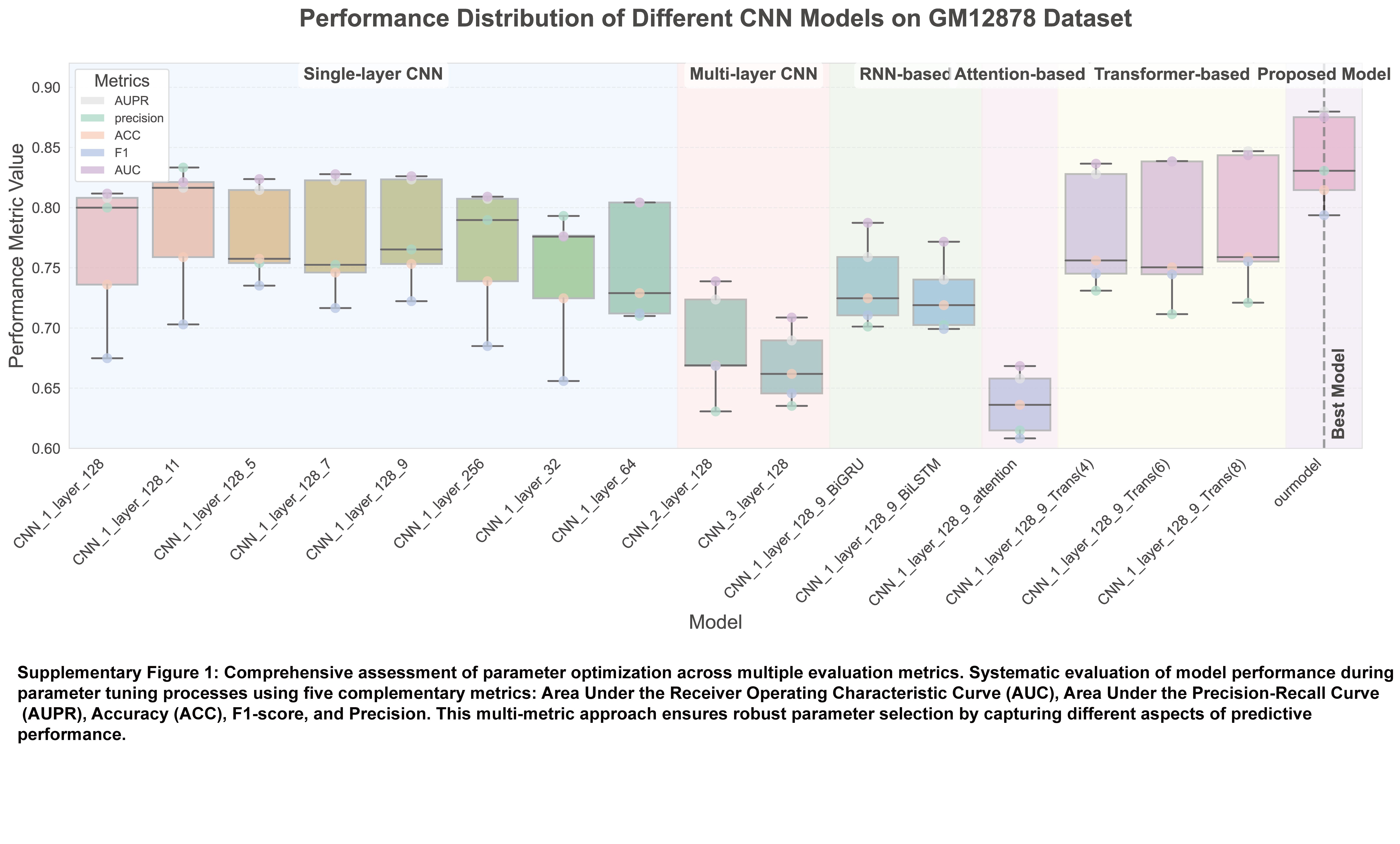

Supplement: Supplementary_Figure_1_bbaf634 [file supplementary_figure_1_bbaf634.jpeg]

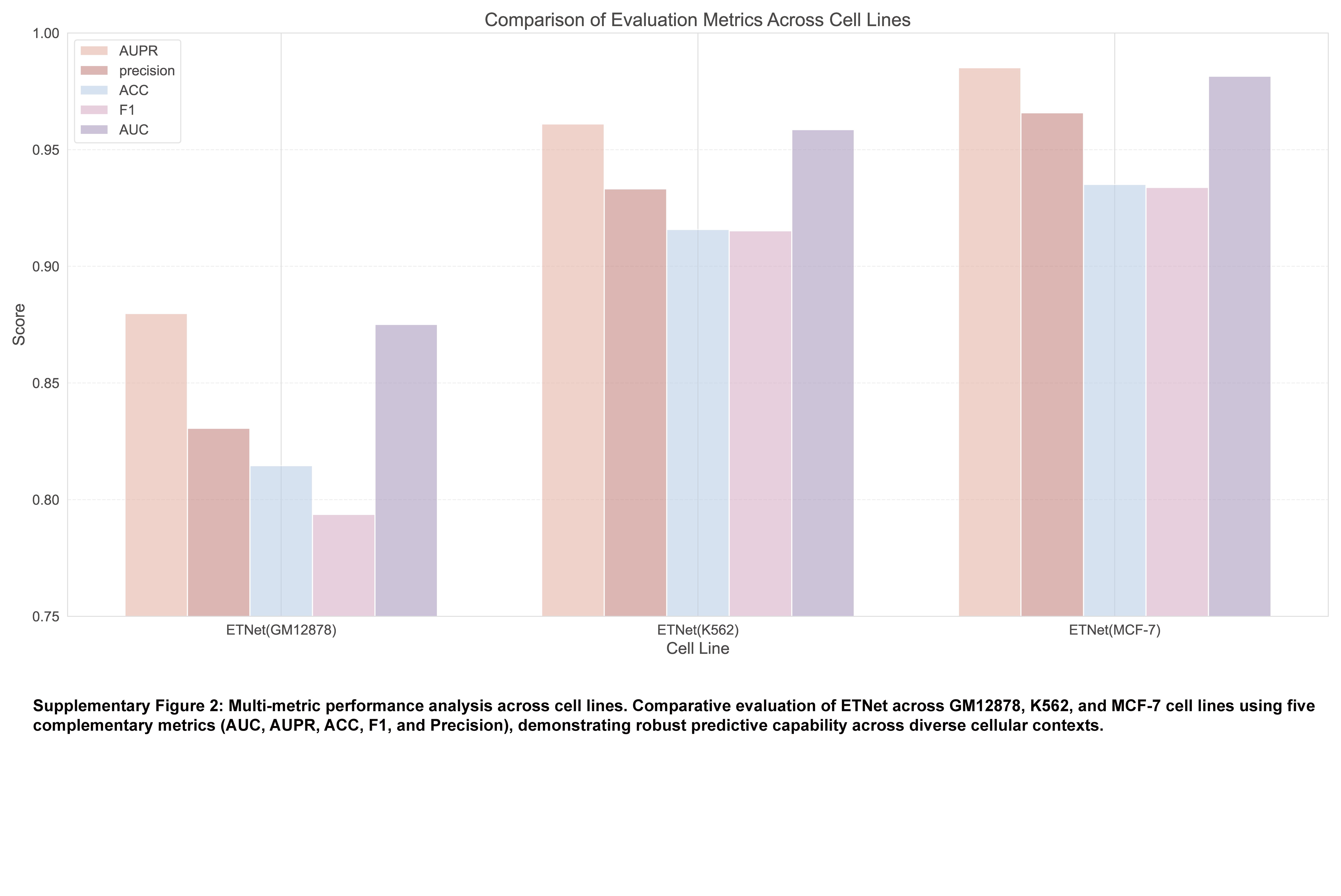

Supplement: Supplementary_Figure_2_bbaf634 [file supplementary_figure_2_bbaf634.jpeg]

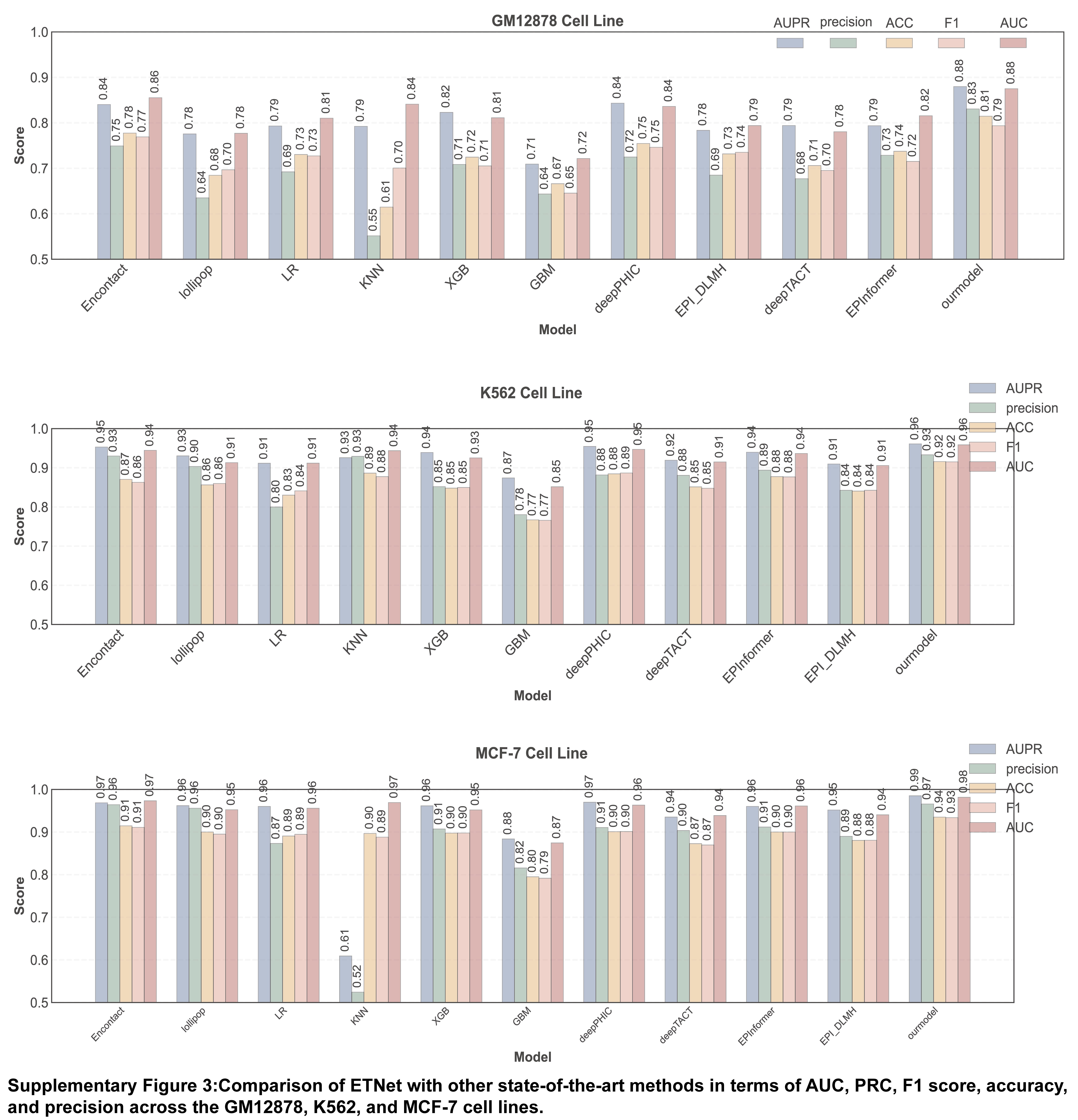

Supplement: Supplementary_Figure_3_bbaf634 [file supplementary_figure_3_bbaf634.jpeg]

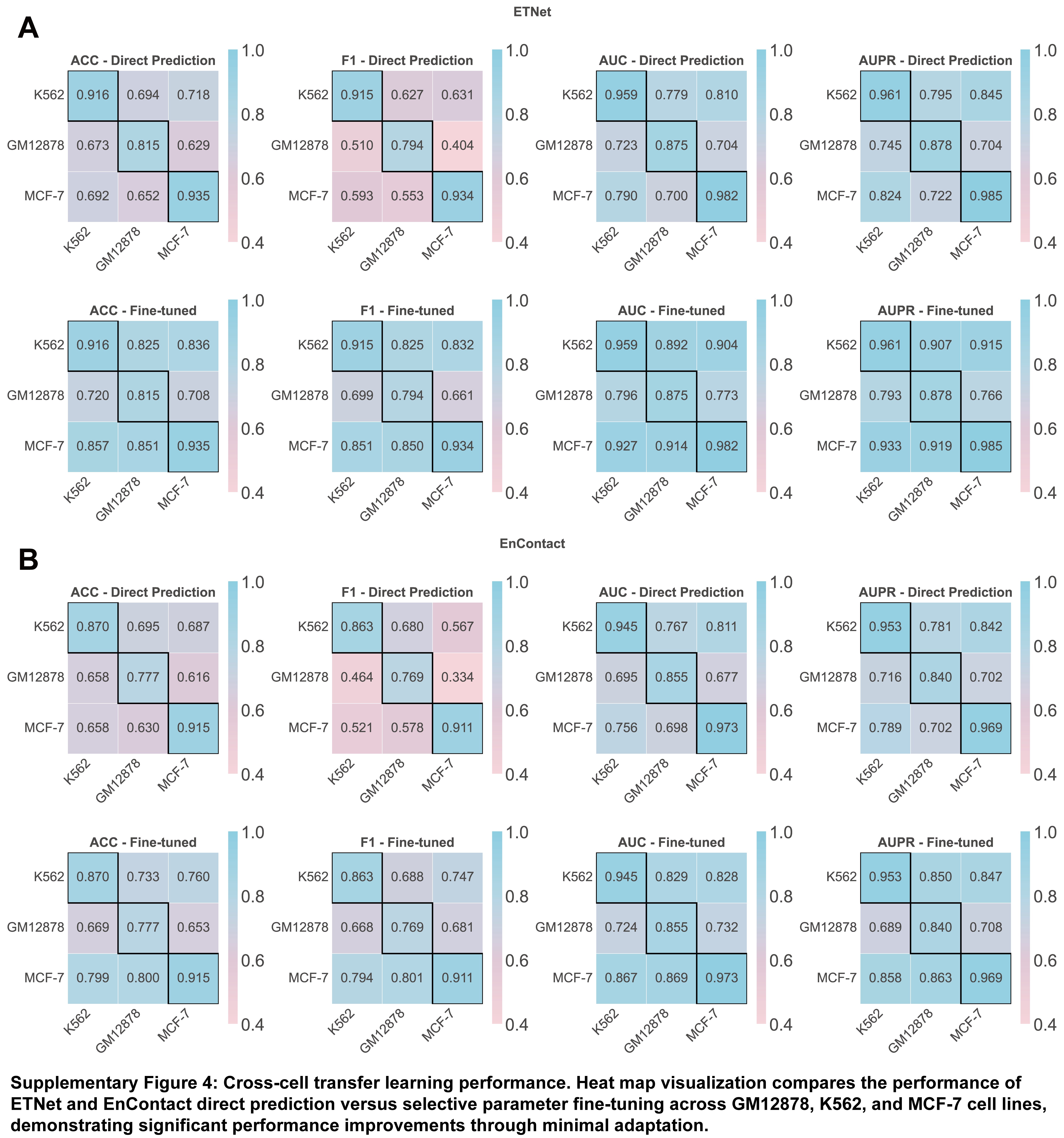

Supplement: Supplementary_Figure_4_bbaf634 [file supplementary_figure_4_bbaf634.jpeg]

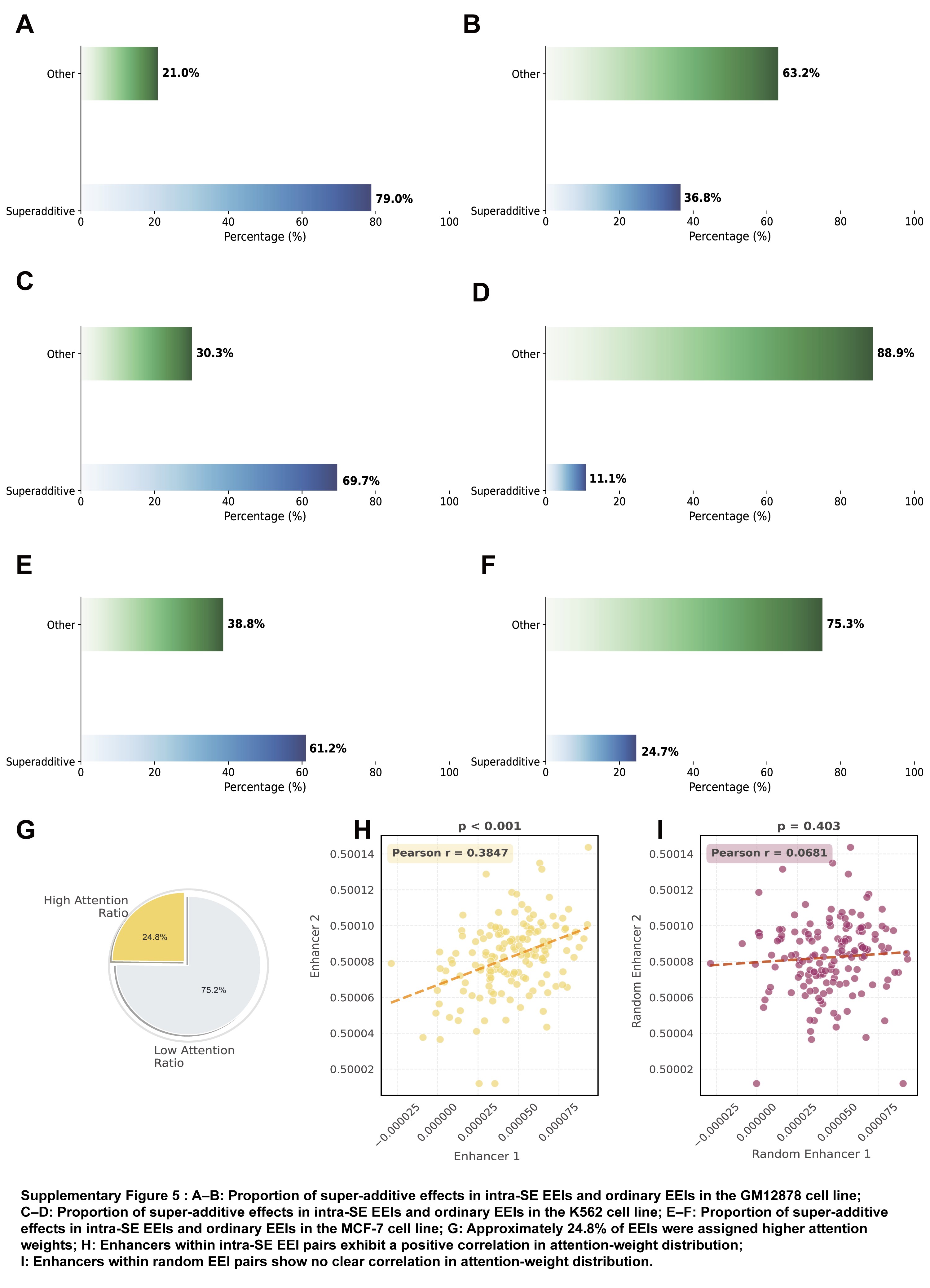

Supplement: Supplementary_Figure_5_bbaf634 [file supplementary_figure_5_bbaf634.jpeg]

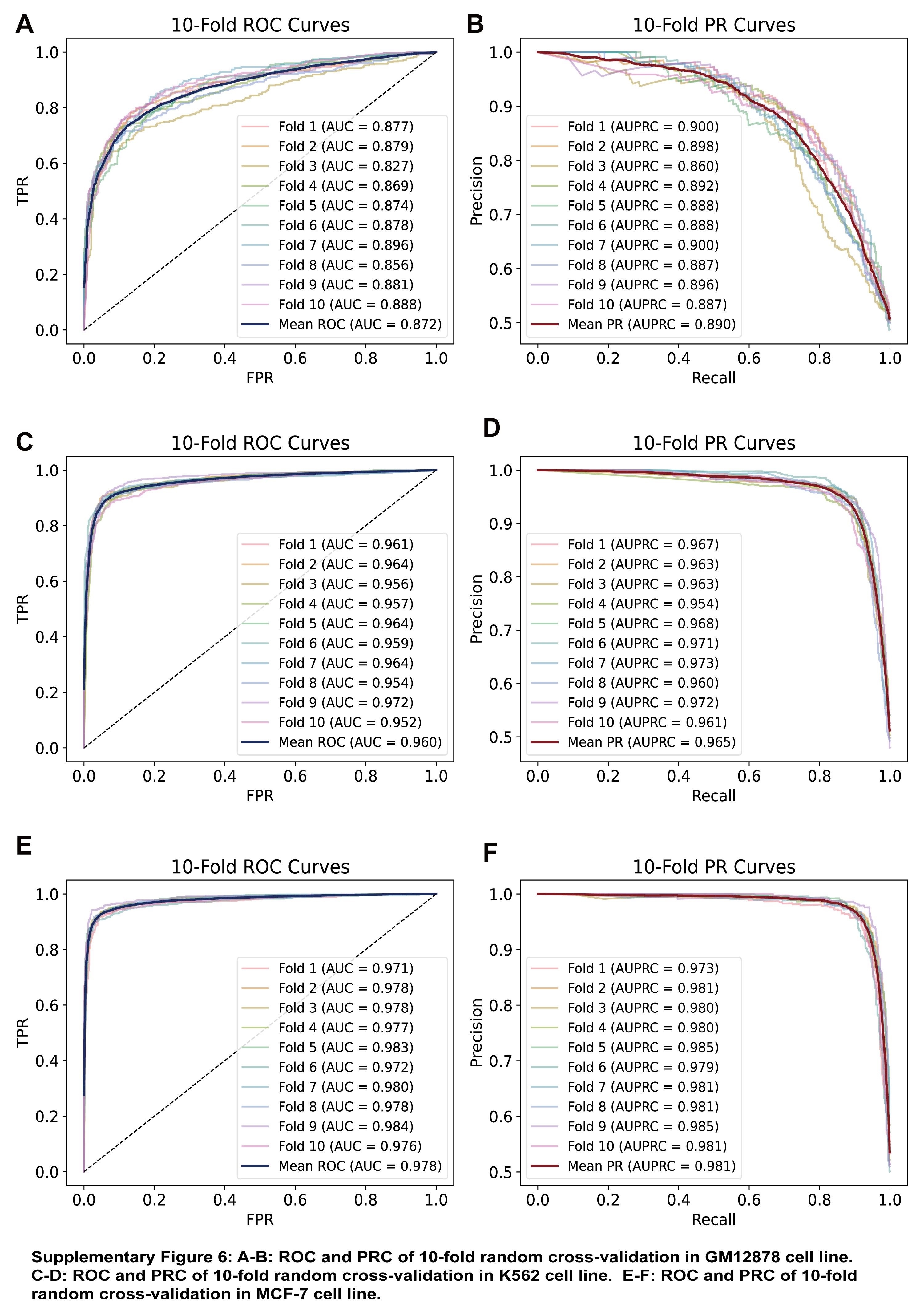

Supplement: Supplementary_Figure_6_bbaf634 [file supplementary_figure_6_bbaf634.jpeg]

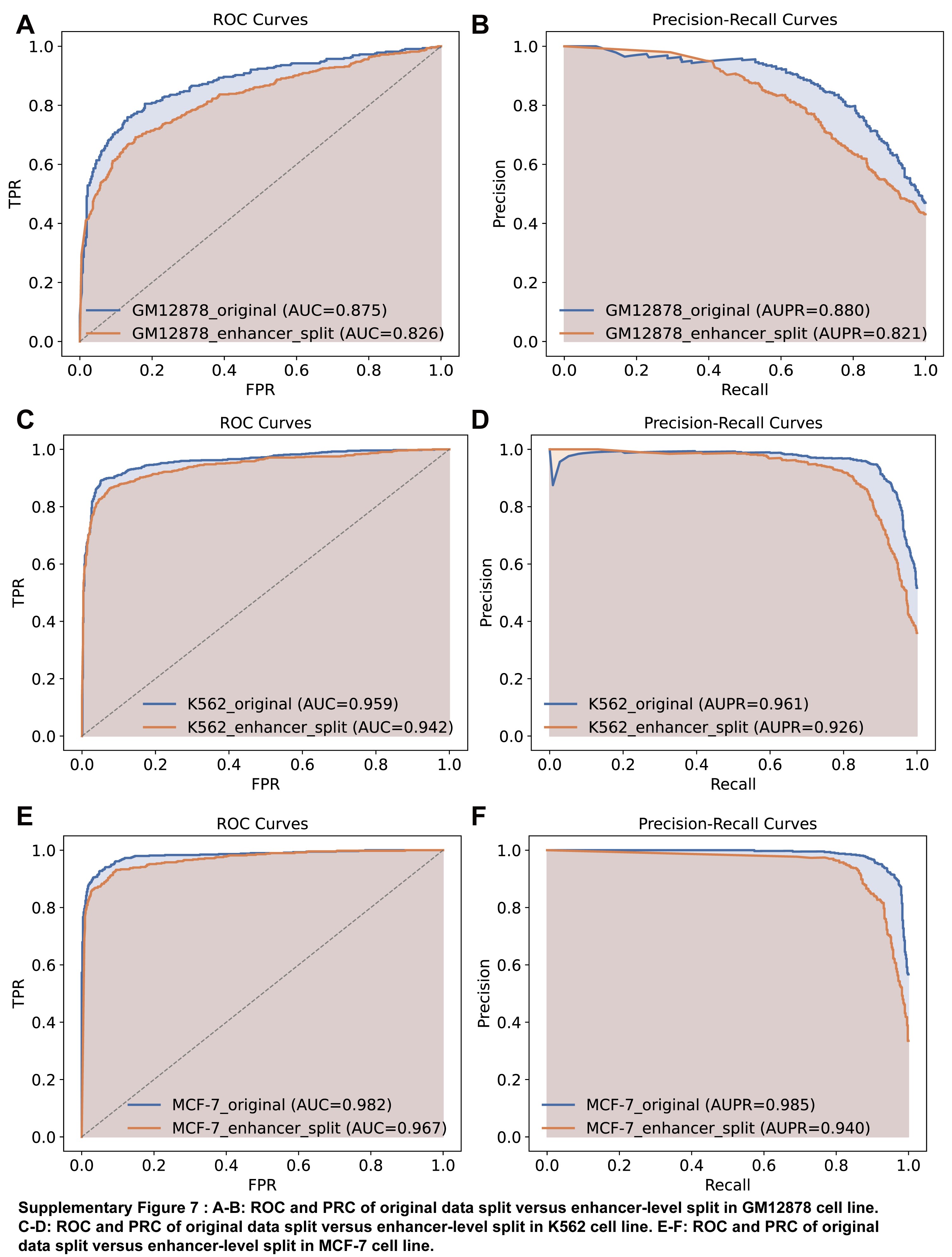

Supplement: Supplementary_Figure_7_bbaf634 [file supplementary_figure_7_bbaf634.jpeg]

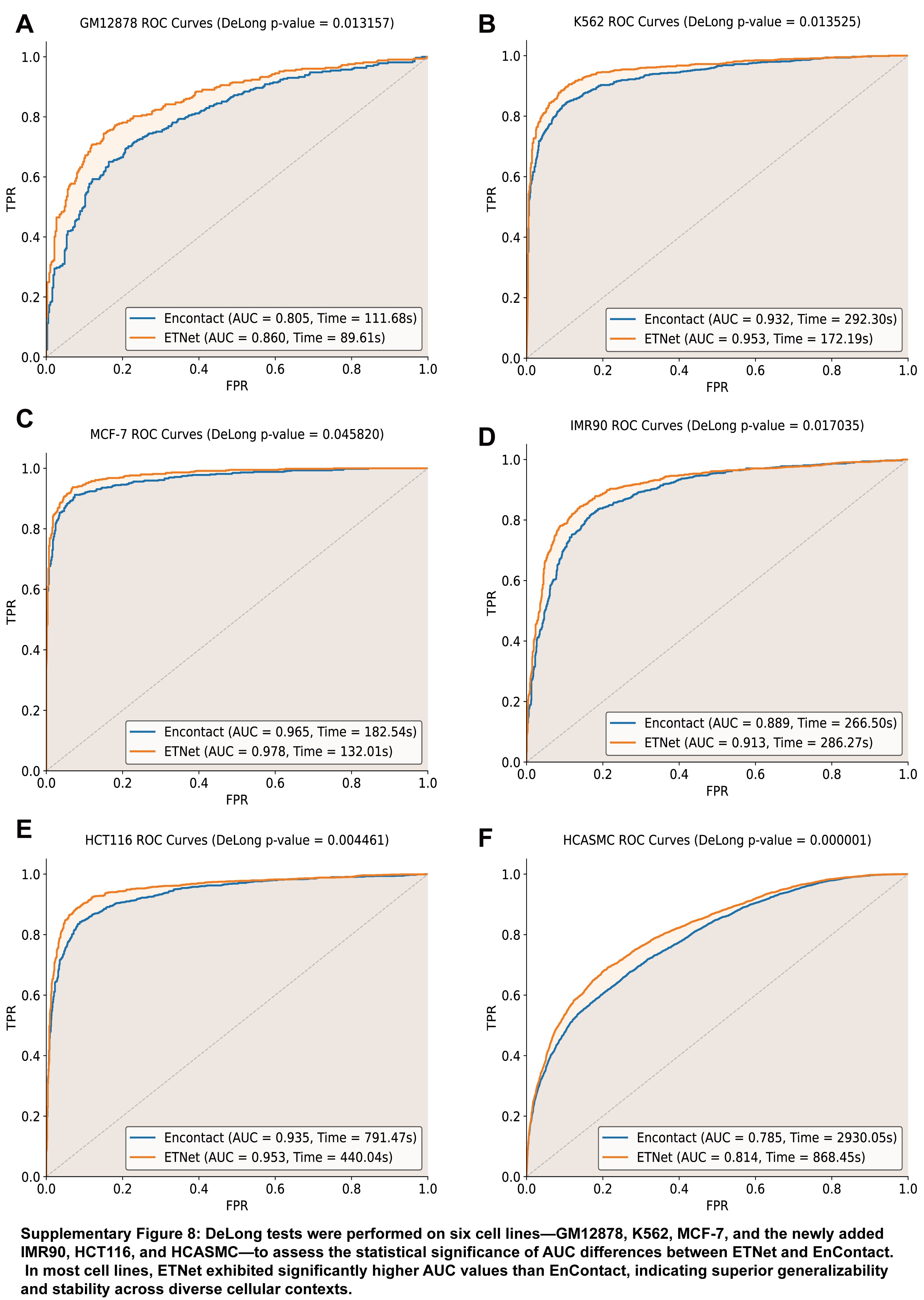

Supplement: Supplementary_Figure_8_bbaf634 [file supplementary_figure_8_bbaf634.jpeg]
