# Supplementary material for: ETNet: an interpretable transformer framework for enhancer–enhancer interaction prediction with cross-context transferability
Source: Brief Bioinform. 2025 Nov 30;26(6):bbaf634. doi: 10.1093/bib/bbaf634 (PMC12665038; doi:10.1093/bib/bbaf634)
Supplement: Supplementary_Tables_bbaf634 [file supplementary_tables_bbaf634.pdf]

Supplementary Table 1. Composition and Distribution of Enhancer-Enhancer Interaction Datasets Across Multiple Cell Lines.

Cell-line	Positive	Negative
GM12878	3506	3506
K562	7853	7853
MCF-7	5882	5882

Supplementary Table 2. ETNet model summary and parameter details.

Layer Type	Output Shape	Param
input_39 (InputLayer)	(None, 2000, 4)	0
input_40 (InputLayer)	(None, 2000, 4)	0
conv1d_18 (Conv1D)	(None, 1000, 128)	4736
conv1d_19 (Conv1D)	(None, 1000, 128)	4736
max_pooling1d_18 (MaxPooling1D)	(None, 500, 128)	0
max_pooling1d_19 (MaxPooling1D)	(None, 500, 128)	0
dropout_93 (Dropout)	(None, 500, 128)	0
dropout_95 (Dropout)	(None, 500, 128)	0
flatten_27 (Flatten)	(None, 64000)	0
flatten_28 (Flatten)	(None, 64000)	0
dense_65 (Dense)	(None, 1000)	64001000
dense_66 (Dense)	(None, 1000)	64001000
dropout_94 (Dropout)	(None, 1000)	0
dropout_96 (Dropout)	(None, 1000)	0
concatenate_19 (Concatenate)	(None, 2000)	0
reshape_29 (Reshape)	(None, 2, 1000)	0
multi_head_attention_9 (MultiHeadAttention)	(None, 2, 1000)	32025000
dropout_97 (Dropout)	(None, 2, 1000)	0
tf.__operators__.add_18 (Add)	(None, 2, 1000)	0
layer_normalization_18 (LayerNormalization)	(None, 2, 1000)	2000
dense_67 (Dense)	(None, 2, 1024)	1025024
dense_68 (Dense)	(None, 2, 1000)	1025000
dropout_98 (Dropout)	(None, 2, 1000)	0
tf.__operators__.add_19 (Add)	(None, 2, 1000)	0
layer_normalization_19 (LayerNormalization)	(None, 2, 1000)	2000
flatten_29 (Flatten)	(None, 2000)	0
dropout_99 (Dropout)	(None, 2000)	0
dense_69 (Dense)	(None, 1)	2001

Supplementary Table 3. Composition and Distribution of Enhancer-Promoter Interaction Datasets Across Multiple Cell Lines.

Cell-line	Positive	Negative
GM12878	2113	2113
K562	1977	1977

Supplementary Table 4. Performance Evaluation of ETNet in Transfer to Enhancer-Promoter Interaction Prediction Task.

Cell-line	AUC	F1	ACC
GM12878	0.8611	0.7934	0.7915
K562	0.8971	0.8549	0.8582

Supplementary Table 5. SNP identification in JAK-STAT pathway. MH and colleagues identified six SNPs (rs560898780, rs1257658099, rs370669851, rs571421696, rs910130021, and rs138606888) located in the enhancer regions of key genes (IRF3, IL7R, JAK2, JAK3, SOCS1, and PTPN2) at different levels of the

SNP ID	Chr	Position(CRCH37)	Variant Type	Alleles	Gene	Anchor1(150 bp upstream)	Anchor2	F_predict	A_predict
rs560898780	chr19	50169275	SNV	G>A	IRF3	chr19:50169125-50169275	chr19:573434-573573	0.9953	0.9933
rs1257658099	chr5	35853936	SNV	C>A	IL7R	chr5:35853786-35853936	chr5:114848957-114849251	0.0015	0.0019
rs571421696	chr19	17962051	SNV	G>A	JAK3	chr19:17961901-17962051	chr19:2330710-2331138	0.002	0.0024
rs370669851	chr9	4983706	SNV	T>C	JAK2	chr9:4983556-4983706	chr9:2018914-2019279	0.27	0.1756
rs910130021	chr16	11351479	SNV	G>T	SOCS1	chr16:11351329-11351479	chr16:67278264-67278626	0.9828	0.9918
rs138606888	chr18	12886059	SNV	G>A	PTPN2	chr16:12886209-12886059	chr18:4983556-4983706	0.0432	0.0523

Supplementary Table 6. Test set data of GM12878, K562, and MCF-7 cell lines before and after deduplication.

Cell-line	Before deduplication	After deduplication
GM12878	1404	1159
K562	3134	2199
MCF-7	2342	1595

Supplementary Table 7. Comparison of ETNet with other methods in AUC, PRC, ACC, and F1 across three cell lines.

		GM12878		
Cell lines	AUC	PRC	ACC	F1
EnContact	0.8553	0.8403	0.7775	0.7692
deepPHiC	0.8362	0.8433	0.7546	0.7463
EPI_DLMH	0.794	0.7835	0.7318	0.7352
deepTACT	0.7805	0.7941	0.7061	0.6953
EPInformer	0.8156	0.7937	0.7375	0.7152
Lollipop	0.777	0.7756	0.6847	0.6968
LR	0.8103	0.7931	0.7304	0.7273
KNN	0.8411	0.7923	0.6148	0.7007
XGB	0.8112	0.823	0.7247	0.7053
GBM	0.7216	0.7094	0.6662	0.6455
**ETNet**	**0.8751**	**0.8798**	**0.8146**	**0.7937**

		K562		
Cell lines	AUC	PRC	ACC	F1
EnContact	0.9445	0.953	0.8705	0.8629
deepPHiC	0.947	0.9545	0.8845	0.8867
EPI_DLMH	0.9058	0.9097	0.8405	0.8426
deepTACT	0.9149	0.9193	0.8513	0.8478
EPInformer	0.9365	0.9397	0.8775	0.8768
Lollipop	0.9131	0.9304	0.8564	0.8599
LR	0.9118	0.9116	0.8302	0.8411
KNN	0.9437	0.9258	0.8864	0.8774
XGB	0.9253	0.939	0.8481	0.8497
GBM	0.8515	0.874	0.7671	0.7659
**ETNet**	**0.9586**	**0.961**	**0.9158**	**0.9152**

		MCF-7		
Cell lines	AUC	PRC	ACC	F1
EnContact	0.9733	0.9686	0.9146	0.911
deepPHiC	0.9632	0.9699	0.9009	0.9012
EPI_DLMH	0.9404	0.9517	0.8804	0.8807
deepTACT	0.9387	0.9352	0.8728	0.8696
EPInformer	0.9612	0.9601	0.9001	0.9001
Lollipop	0.9523	0.9619	0.9001	0.8951
LR	0.9558	0.96	0.8907	0.8947
KNN	0.9692	0.6092	0.8967	0.8881
XGB	0.9519	0.9615	0.8975	0.8978
GBM	0.8745	0.8837	0.795	0.7917
**ETNet**	**0.9815**	**0.9851**	**0.9351**	**0.9338**

Supplementary Table 8. Composition and Distribution of Enhancer-Enhancer Interaction Datasets Across Multiple Cell Lines.

Cell-line	Positive	Negative	sources
IMR90	10000	10000	**GSE86189**
HCT116	12969	12969	**GSE161873**
HCASMC（Human coronary artery smooth muscle cells）	10000	10000	**https://www.nature.com/articles/ng.3963**
